# Development of a brief assessment tool to identify children with probable anxiety disorders

**DOI:** 10.1002/jcv2.12265

**Published:** 2024-08-17

**Authors:** Tessa Reardon, Obioha C. Ukoumunne, Susan Ball, Paul Brown, Tamsin Ford, Alastair Gray, Claire Hill, Bec Jasper, Michael Larkin, Ian Macdonald, Fran Morgan, Michelle Sancho, Falko F. Sniehotta, Susan H. Spence, Jason Stainer, Paul Stallard, Mara Violato, iCATS Team, Cathy Creswell

**Affiliations:** ^1^ Departments of Experimental Psychology and Psychiatry University of Oxford UK; ^2^ NIHR ARC South West Peninsula (PenARC) Department of Health and Community Sciences Faculty of Health and Life Sciences University of Exeter UK; ^3^ Bransgore C of E Primary School UK; ^4^ University of Cambridge and Cambridge and Peterborough Foundation Trust UK; ^5^ Health Economics Research Centre Nuffield Department of Population Health University of Oxford UK; ^6^ School of Psychology & Clinical Language Sciences University of Reading UK; ^7^ Parents and Carers Together Suffolk UK; ^8^ Life and Health Sciences Aston University Birmingham UK; ^9^ Charlie Waller Trust UK; ^10^ Square Peg UK; ^11^ West Berkshire Council Newbury UK; ^12^ NIHR Policy Research Unit Behavioural Science Newcastle University UK; ^13^ Division of Public Health Social and Preventive Medicine Center for Preventive Medicine and Digital Health (CPD) Universitätsmedizin Mannheim Heidelberg University Heidelberg Germany; ^14^ School of Applied Psychology and Australian Institute of Suicide Research and Prevention Griffith University Australia; ^15^ Stanley Primary School London UK; ^16^ Department of Health University of Bath UK; ^17^ Oxford NHS Foundation Trust UK

**Keywords:** anxiety, brief measure, children, identification, screening

## Abstract

**Background:**

Difficulties identifying anxiety disorders in primary‐school aged children present significant barriers to timely access to support and intervention. This study aimed to develop a brief assessment tool that can identify children with anxiety disorders in community settings, with a high level of sensitivity and specificity.

**Methods:**

Children (aged 8–11 years), and their parents/carers and teachers from 19 primary/junior schools in England each completed a pool of questionnaire items that assessed child anxiety symptoms and associated impact. Diagnostic assessments (Anxiety Disorder Interview Schedule for Children: Child and Parent interviews) were administered by independent assessors to determine the presence/absence of anxiety disorders in children. We created alternative candidate brief child‐, parent‐, teacher‐report questionnaires consisting of the ‘best’ items selected from the wider pool of completed items. We used exploratory factor analysis to reduce the item pool, and multivariable backward elimination logistic regression to identify items that were the strongest predictors of the presence/absence of an anxiety disorder.

**Results:**

Parents/carers of 646 children provided consent; child/parent/teacher‐report questionnaires were collected for 582/646/565 children respectively; and diagnostic outcome data were collected for 463 children. None of the brief child‐ nor teacher‐report questionnaires achieved acceptable sensitivity/specificity (<75%). Parent‐report questionnaires including between 2 and 9 items that assess anxiety symptoms and/or associated impact achieved acceptable sensitivity and specificity (≥75%).

**Conclusions:**

The two‐item parent‐report measure that assesses distress and impairment associated with anxiety brings the advantage of brevity and has the potential to be used in community settings to improve identification of children with anxiety disorders.

**Key points**

**What's known?**

- Brief questionnaires that can accurately identify children with diagnoseable anxiety disorders in community settings would address key barriers to treatment access.

**What's new?**

- Our findings show that brief parent‐report questionnaires (2–9 items) can identify children (8–11 years) with anxiety disorders in primary schools with acceptable sensitivity and specificity.

**What's relevant?**

- The two‐item parent‐report questionnaire that assesses impact associated with anxiety symptoms provides an efficient tool for improving identification of childhood anxiety disorders in community settings.

Anxiety disorders are among the most common mental health disorders experienced by primary‐school aged children (NHS Digital, 2018), and are associated with a wide range of negative consequences (Pollard et al., 2023). Psychological interventions are effective for treating childhood anxiety disorders (James et al., 2020), but few children access these interventions (Reardon et al., 2020). Parents report substantial barriers related to identifying anxiety problems in children (Reardon et al., 2020; Reardon, Harvey, et al., 2018), school staff (Department for Education, 2017) and GPs (O’Brien et al., 2019) lack confidence in identifying common mental health problems in children, and teachers often fail to identify children with anxiety problems (Mathews et al., 2021). Approaches to improve the identification of anxiety disorders in children are needed to minimise these key barriers to treatment access.

Systematic screening is one approach to improve identification of under‐ or mis‐diagnosed illnesses. The US Preventive Services Task Force recently recommended screening for anxiety disorders among 8–18 year olds (Mangione et al., 2022). Schools provide an ideal setting for assessing all children (Dowdy et al., 2015) and are used for physical health checks in the UK (NHS Digital, 2022). Although both school staff and parents identify potential benefits of universal screening for common mental health problems, there are concerns related to the accuracy of screening results and the time and resource required to administer screening questionnaires (Childs‐Fegredo et al., 2021; Soneson et al., 2018). Consequently, few schools use this approach (e.g. NatCen Social Research, 2017). The UK National Screening Committee specify the availability of a ‘simple, safe, precise and validated screening test’, with a ‘suitable cut‐off level defined and agreed’ as prerequisites for screening (UK National Screening Committee, 2022), and such a test does not currently exist for childhood anxiety disorders.

Existing child anxiety questionnaire measures are typically available in child‐ and parent‐report versions and include about 40 questions that assess symptoms associated with anxiety disorders (e.g., Spence Children's Anxiety Scale (SCAS), Revised Children's Anxiety and Depression Scale (RCADS), Screen for Child Anxiety Related Disorders (SCARED)). The SCAS and the RCADS (which is partially derived from the SCAS) bring advantages that are particularly pertinent in the context of screening where concerns related to time and resource burden are common: both are freely available and initial evaluations of the reliability and validity of shortened versions are promising (Ebesutani et al., 2012, 2017; Reardon, Spence, et al., 2018). Encouragingly, as few as 8 items from the parent‐, child‐, and teacher‐reported SCAS can discriminate between a community and clinic‐referred sample of children aged 7–11 years with anxiety disorders (Reardon, Spence, et al., 2018). However, we do not know whether these short (or longer) questionnaires can accurately discriminate between primary‐school aged children with and without anxiety disorders *within a community* sample. A recent review of diagnostic accuracy studies that have evaluated anxiety screening tools in community samples of participants ≤18 years (Viswanathan et al., 2022) identified 10 eligible studies that evaluated 12 measures and reported variable accuracy (sensitivity: 34%–100%; specificity: 47%–99%). However, the only included study that reported specifically on accuracy among children ≤12 years focussed specifically on social phobia (Bailey et al., 2006). As such it is difficult to draw conclusions about the potential capacity of anxiety symptom questionnaire measures completed by children, parents or others to identify primary school‐aged children with any anxiety disorder.

Focussing exclusively on anxiety *symptoms* may not be the optimal approach to identifying children with/without anxiety *disorders*. Anxiety disorder diagnoses are characterised by the presence of specific symptoms *and* associated distress or impairment (American Psychiatric Association, 2013), and only assessing symptoms in community samples may increase the risk of false positives (Rapee et al., 2012). Questionnaire measures of the impact of anxiety symptoms developed for clinical populations (e.g. Child Anxiety Impact Scale (CAIS) (Langley et al., 2004, 2014)) are valued by families (Creswell et al., 2021), and are better predictors of recovery from child anxiety disorders in clinical samples than symptom measures (Evans et al., 2017). Notably, items from the Strengths and Difficulties Questionnaire (SDQ) ‘impact supplement’ that assesses impairment associated with emotional and behavioural problems are better able to discriminate a psychiatric clinic sample from a community sample than symptom items alone (Goodman, 1999). Furthermore, using a combination of symptom items followed by ‘impact’ items is optimal for identifying children with mental health disorders within community samples (Goodman et al., 2000). However, the capacity of items specifically designed to assess impact associated with *anxiety* symptoms (either alone or in combination with anxiety symptom items) has not been evaluated for screening purposes.

This study aimed to develop an assessment tool to identify primary school children with anxiety disorders. We set out to: 1) develop brief and acceptable questionnaire measures (child‐, parent‐, teacher‐report) that can accurately identify children with and without anxiety disorders; 2) establish a cut‐off score on each questionnaire (child‐, parent‐, teacher‐report) that can detect children with anxiety disorders with a high level of sensitivity and specificity; 3) identify the optimal single or combination of reporters that can most accurately identify children with anxiety disorders.

## METHODS

### Study design

Children (aged 8–11 years), their parents/carers and class teachers each completed a large pool of questionnaire items to be considered for inclusion in the brief child anxiety questionnaires (index test). The item pool included items that assess anxiety symptoms and associated impact, and included a combination of items from existing measures and new items. Independent assessors then administered separate structured diagnostic interviews (reference standard) with children and their parents/carers to determine the presence/absence of a child anxiety disorder. We then selected the ‘best’ items from the wider pool of completed questionnaire items to form new ‘candidate’ brief measures, and assessed the accuracy of these candidate measures.

We recruited participants from mainstream primary and junior schools in England, and recruitment and data collection took place between October 2019 and July 2020. The study was approved by the University of Oxford Medical Sciences Interdivisional Research Ethics Committee (Reference: R64592) and the study protocol was registered on the Open Science Framework (https://osf.io/y7na6).

### Sample size

Our original recruitment target was 770 children. We anticipated that to achieve this we would need to recruit approximately 22 schools (based on an estimated response rate of 20% and 180 eligible children per school). Based on an estimated prevalence rate of 6.5% (Polanczyk et al., 2015), we anticipated 50 out of 770 children would have an anxiety disorder diagnosis on the basis of the diagnostic assessment. Although there are no clearly defined and agreed levels of sensitivity and specificity required for school‐based screening, a minimum of 75%–85% for both is often recommended (Glover & Albers, 2007). We therefore considered sensitivity and specificity of ≥85% as optimal, and ≥75% as acceptable. Fifty children with an anxiety disorder is sufficient to estimate a sensitivity of 85% with a standard error of 5 percentage points and 720 children without an anxiety disorder is enough to estimate a specificity of 85% with a standard error of 1.3 percentage points (see Appendix 1 for further detail on the sample size justification).

Data collection coincided with the COVID‐19 pandemic and school closures from March to July 2020. Our original target sample size was 770 children on the basis that this would include at least 50 children who met diagnostic criteria for an anxiety disorder. At the point where schools closed in March 2020, our sample already included more than 50 children with an anxiety disorder. Given the substantial disruption and uncertainty for schools and families at this time, we adjusted procedures to allow us to continue with outstanding data collection in participating schools remotely, but did not recruit new participants in any further schools after March 2020.

### Recruitment and data collection

**FIGURE 1:**
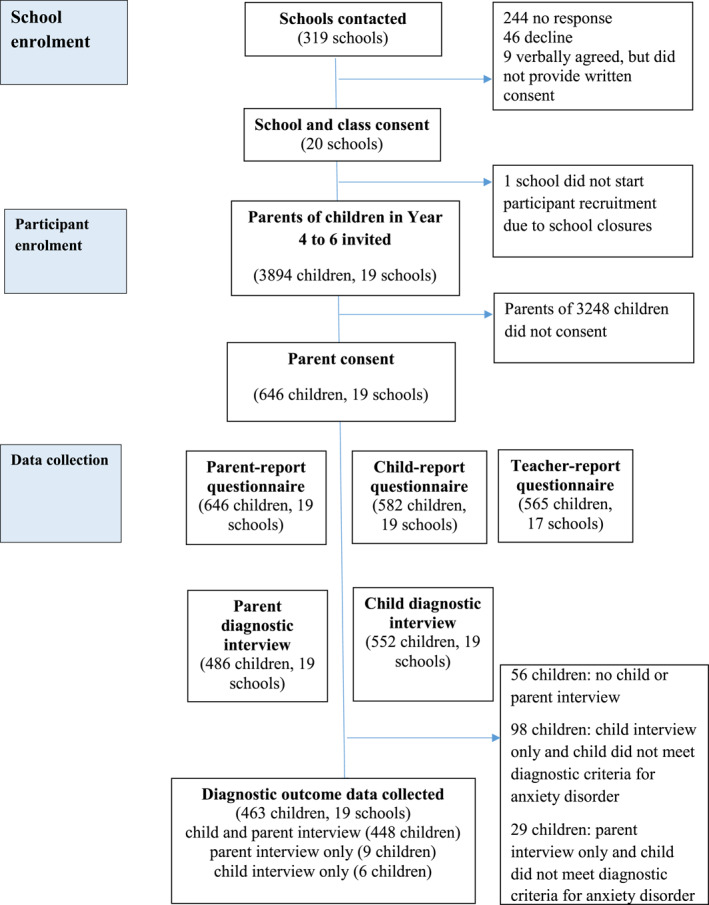
Participant flow.

We contacted 319 primary/junior schools in the South of England, and 19 schools participated (see Figure 1). Participating schools were from seven local authority areas and varied in size (241–679 pupils on the roll) and level of deprivation (2.5%–32.7% pupils eligible for free school meals).

Parents/carers (hereby referred to as parents) of all children in Year 4–Year 6 in participating schools were invited to take part and to provide written consent for their child/ren to participate. Where parents provided written consent, children were invited to participate and written assent was obtained from children before collecting any child‐report measures.

Parents completed questionnaires in their own time on paper or online (all prior to school closures in March 2020). Before COVID‐19 school closures, researchers visited schools to administer questionnaires with groups of children at school (16 schools) and, once this was not possible, children completed questionnaires at home (3 schools). Class teachers (or a nominated member of staff who works regularly with the child) completed teacher‐report questionnaires in their own time online or on paper.

Diagnostic interviews with parents were administered by telephone, and (prior to 20 March 2020) face‐to‐face with children at school or (after 20 March 2020) via video or phone call, within 12 weeks of questionnaire collection.

### Measures

#### Child anxiety questionnaire items (child‐, parent‐ and teacher‐report versions)

The pool of child anxiety questionnaire items completed by participants (66 child‐report items, 73 parent‐report items, 62 teacher‐report items) included items that assessed 1) symptoms of DSM‐5 anxiety disorders and 2) associated impact (including impact, chronicity, and perceived need for help). All items were rated on a four‐point ordinal scale (scored 0–3). Participants were also given the opportunity to provide written feedback on items.

Symptom items consisted of items from the SCAS, items from the RCADS‐Anxiety Scale that are not included on the SCAS, and new items developed by the researchers that assess anxiety symptoms not addressed in either the SCAS or the RCADS. Responses to relevant SCAS/RCADS items were used to calculate SCAS‐8 total scores (Reardon, Spence, et al., 2018; possible scores 0–24) and RCADS‐25‐Anxiety Scale total scores (Ebesutani et al., 2012, 2017; possible scores 0–45) for each reporter to provide comparative short child anxiety measures (see below).

Items to assess impact associated with anxiety symptoms were new items developed by the research team that draw on the content of items from relevant existing measures, including the SDQ impact supplement, Child Anxiety Impact Scale and Child Anxiety Life Interference Scale. Wording of items reflected the reporter, and items related to impact on the parent's life were only included in the parent‐report version. Further details about the anxiety items and relevant existing measures are provided in Appendix 2.

#### Anxiety Disorder Interview Schedule child version: Child and parent interviews (ADIS‐C/P)

The ADIS‐C/P was used to determine the presence/absence of child anxiety disorder diagnoses (reference standard), and common comorbid disorders. Separate child and parent interviews were administered by a team of trained assessors who were blind to child/parent/teacher questionnaire responses. Standard interview schedules developed for DSM‐IV were followed, with minor amendments to interviews to enable diagnostic outcomes consistent with the DSM‐5. As per standard guidance, diagnoses and Clinical Severity Ratings (CSRs) four to eight were assigned if the child met diagnostic criteria on the basis of *either* the child *or* the parent interview. The diagnosis with the highest CSR was assigned the primary diagnosis.

For each assessor, diagnoses and CSRs for at least the first 20 child interviews and first 20 parent interviews were double rated by a consensus team led by an experienced diagnostician (TR). After these initial interviews, and once assessors achieved high inter‐rater reliability with the consensus team (minimum Kappa statistic for presence/absence of diagnoses = 0.85; minimum intra‐class correlation coefficient (ICC) for CSRs = 0.85), a minimum of one in six interviews for each assessor were double rated. Overall, inter‐rater reliability for all double rated assessments was excellent (Kappa = 0.86 for diagnosis, and ICC = 0.91 for CSR).

For the main analyses, children were classified as ‘anxiety diagnosis’ if they were assigned a diagnosis with a CSR four to eight for at least one anxiety disorder, and ‘no anxiety diagnosis’ if they were not assigned any anxiety disorder diagnosis with a CSR 4–8 (see Appendix 2 for further details).

#### Additional measures and information

Children, parents, and teachers each completed the 25‐item SDQ, and responses to the 5‐item SDQ‐Emotional subscale (SDQ‐E; possible total score range 0–10) were used to provide a comparative short symptom measure (see below). We collected socio‐demographic information from parents and the child's school record.

### Analysis

We created alternative candidate brief child‐, parent‐, and teacher‐report questionnaires that each comprised of a selection of the ‘best’ items chosen from the wider pool of completed items. As mental health disorder symptoms and functional impairment differ conceptually (Rapee et al., 2012), and we know little about the extent to which either or both need to be assessed to accurately identify anxiety disorders in community populations, we created three types of candidate measures for each reporter: 1) ‘symptom/impact’ measures that comprise of items selected from the whole pool of completed symptom and impact items; 2) separate ‘symptoms’ and ‘impact’ measures to be used in a ‘two‐stage’ screen, where children are first screened for symptoms and those with a positive result are then screened for associated impact, with items selected separately for each stage; and 3) ‘impact only’ measures that comprise of selected impact items. The candidate measures each produced quantitative scores (sum of item scores), with an appropriately derived cut‐point to discriminate between children with and without an anxiety disorder. The criterion for evaluating the candidate measures was their ability to discriminate between children who were classified as ‘anxiety diagnosis’ and ‘no anxiety diagnosis’ on the basis of the diagnostic assessment (ADIS–C/P). To facilitate comparison with existing measures, we used responses to relevant items to also evaluate the capacity of existing short‐symptom based measures (SCAS‐8, RCADS‐25‐Anxiety Scale, SDQ‐E) to discriminate children with and without anxiety disorders.

#### Item selection for candidate measures

##### Item selection for candidate symptom/impact measures

First, we reviewed summaries of item responses and participant feedback to identify whether there were any items that may not be useful (e.g. very little variability, poorly understood) and should be removed from the outset. Next, to reduce the item pool to a manageable number, we used exploratory factor analysis to identify the most salient factors underlying the items and selected the item with highest loading for each factor. For each reporter, exploratory factor analysis models were fitted using all symptom and impact items. We used the parallel analysis method (Brown, 2015) where data simulation was used to determine the number of salient factors, and these were rotated using an oblique rotation method (*geomin*). We then fitted backward elimination multivariable logistic regression models using the items with the highest loading on each factor as predictors, and anxiety disorder status as outcome. For these analyses, items were treated as continuous predictors, and items with a *p*‐value greater than 0.05 were removed from the model. The set of items in the final model comprised provisional items for inclusion in a candidate screening measure. To confirm we had not omitted any important items, we then considered each item that had been previously discarded and added them (one at a time) to a multivariable logistic regression model with the other provisional items. Where there was an indication of a marked improvement in prediction of anxiety disorder status (defined as an increase of at least 0.01 in the area under the curve, AUC), the previously discarded item that produced the largest increase in the AUC was included in the candidate screening measure. The process was continued until there were no further items for which their inclusion resulted in an increase of at least 0.01 in the AUC. Finally, the research team (including clinicians with expertise in child anxiety, and parents with lived experience of child anxiety disorders) reviewed the face validity of the selected set of items as a stand‐alone short measure and where there were concerns, we replaced individual items with alternative items to provide an alternative candidate measure.

##### Item selection for separate candidate ‘symptoms’ and ‘impact’ measures

To select the ‘best’ items from the pool of symptom items, we ran exploratory factor analysis as above but only including symptom items and selected the items with the highest loading on each ‘symptoms’ factor. We then followed the same multivariable backward elimination logistic regression approach and consideration of disregarded items as above to produce a candidate ‘symptoms’ screening measure. As before, we also reviewed the face validity of selected items and where there were concerns, replaced individual items with alternative items.

The pool of interference items was smaller than the symptom item pool so we did not use factor analysis to reduce the impact item pool. Instead, we selected impact items considered to have strong face validity for use in a brief measure, and included these as predictors in backward elimination logistic regression models to identify items that were the strongest predictors of diagnostic status among children who scored at least one on the candidate ‘symptoms’ measure.

#### Evaluation of candidate measures

For each reporter (child, parent, teacher), we used receiver operating characteristic (ROC) analyses to identify optimal cut‐off scores for each candidate measure.

For the candidate symptom/impact measures including items selected from the whole item pool (candidate measure 1), item scores were summed to produce a total score, and we identified the optimal cut‐off total score and reported the associated sensitivity and sensitivity.

For symptom measures, item scores were summed to produce a total ‘symptoms’ score, and impact measure items were summed to produce a total ‘impact’ score. We then identified optimal cut‐off total scores for two‐stage screens, using a symptom measure (Stage 1) and impact measure (Stage 2) in sequence (candidate measure 2), and using the impact only measure (candidate measure 3).

Where an acceptable level of accuracy was achieved (≥75% sensitivity and specificity) for a candidate measure, we explored characteristics of ‘false positives’ and examined measure performance across subgroups based on gender, year group and ethnicity. To facilitate comparison with existing measures, we also examined sensitivity/specificity for the SCAS‐8, RCADS‐25‐Anxiety Scale and SDQ‐E for each reporter.

If more than one single reporter achieved an acceptable level of accuracy, we also set out to examine the sensitivity/specificity values associated with using a combination of reporters, on the basis that this could improve sensitivity, without a marked reduction in specificity.

## RESULTS

### Participants

Figure 1 displays participant flow through the study and Table 1 summarises participant characteristics.

**TABLE 1:** Sample characteristics.

	Total (*N* = 646)	Children with diagnostic outcome data (*N* = 463)
Year group		
Year 4, *n* (%)	222 (34.4)	153 (33.0)
Year 5, *n* (%)	242 (37.5)	173 (37.4)
Year 6, *n* (%)	182 (28.2)	137 (29.6)
Child age, mean (SD)	9.81 (0.83), *N* = 645	9.83 (0.83), *N* = 463
Child gender		
Female, *n* (%)	333 (51.5)	243 (52.5)
Male, *n* (%)	312 (48.3)	220 (47.5)
Missing, *n* (%)	1 (0.2)	0 (0)
Child ethnicity		
White British, *n* (%)	524 (81.3)	378 (81.6)
Other ethnic background, *n* (%)	118 (18.3)	85 (18.4)
Not stated, *n* (%)	3 (0.5)	0 (0)
Missing, *n* (%)	1 (0.2)	0 (0)
Parent gender		
Female, *n* (%)	583 (90.2)	419 (90.5)
Male, *n* (%)	62 (9.6)	44 (9.5)
Missing, *n* (%)	1 (0.2)	0 (0)
Parent age, mean (SD)	40.0 (5.9), *N* = 619	40.4 (5.69), *N* = 446
Parent education		
School completion, *n* (%)	81 (12.5)	44 (9.5)
Further education (college/vocational), *n* (%)	244 (37.8)	162 (35.0)
Higher education (undergraduate), *n* (%)	185 (28.6)	149 (32.2)
Postgraduate qualification, *n* (%)	103 (15.9)	84 (18.1)
Missing, *n* (%)	33 (5.1)	24 (5.2)
Type of housing		
Rented, *n* (%)	216 (33.4)	137 (29.6)
Mortgage/fully owned, *n* (%)	386 (60.0)	298 (64.4)
Other, *n* (%)	19 (2.9)	10 (2.2)
Missing, *n* (%)	25 (3.9)	18 (3.9)
Multiple index of deprivation decile (1 = most deprived, 10‐least deprived),		
Median (range; inter‐quartile range)	7 (1–10; 5–9)	7 (1–10; 5–9)
Child's eligibility for free school meals		
Eligible, *n* (%)	76 (11.8)	43 (9.3)
Not eligible, *n* (%)	567 (87.8)	419 (90.5)
Missing *n* (%)	3 (0.5)	1 (0.2)
SCAS‐8‐C, mean (SD)	6.3 (4.33), *N* = 579	6.4 (4.35), *N* = 450
SCAS‐8‐P, mean (SD)	6.9 (4.33), *N* = 635	6.9 (4.42), *N* = 455
SCAS‐8‐T, mean (SD)	3.7 (3.04), *N* = 520	3.6 (3.02), *N* = 381
Anxiety disorder status		
Anxiety disorder, *n* (%)	107 (16.6)	107 (23.1)
No anxiety disorder, *n* (%)	356 (55.1)	356 (76.9)
Missing, *n* (%)	183 (28.3)	0 (0)
Presence of specific anxiety disorder diagnosis		
Generalised anxiety disorder, *n* (%)		60 (13.0)
Social anxiety disorder, *n* (%)		27 (5.8)
Separation anxiety disorder, n (%)		32 (6.9)
Specific phobia, *n* (%)		23 (5.0)
Panic disorder, *n* (%)		1 (0.2)
Agoraphobia, *n* (%)		1 (0.2)
Selective mutism, *n* (%)		2 (0.4)
Other specified anxiety disorder, *n* (%)		4 (0.9)
Presence of other disorders		
OCD, *n* (%)		3 (0.6)
Depressive disorder, *n* (%)		3 (0.6)
ADHD/ADD		34 (7.3)
ODD, n (%)		4 (0.9)
Primary disorder diagnosis		
Generalised anxiety disorder, *n* (%)		42 (9.1)
Social anxiety disorder, *n* (%)		16 (3.5)
Separation anxiety disorder, *n* (%)		20 (4.3)
Specific phobia, *n* (%)		8 (1.7)
Panic disorder, *n* (%)		1 (0.2)
Agoraphobia, *n* (%)		1 (0.2)
Selective mutism, *n* (%)		2 (0.4)
Other specified anxiety disorder, *n* (%)		4 (0.9)
OCD, *n* (%)		2 (0.4)
ADHD/ADD, *n* (%)		22 (4.8)
Depressive disorder, *n* (%)		1 (0.2)
ODD, *n* (%)		3 (0.6)
Highest anxiety disorder CSR, n		
CSR 2		11
CSR 3		43
CSR 4		52
CSR 5		38
CSR 6		14
CSR 7		3

Abbreviation: ADD, Attention Deficit Disorder; ADHD, Attention Deficit Hyperactivity Disorder; CSR, Clinical Severity Rating; OCD, Obsessive Compulsive Disorder; ODD, Opposition Defiant Disorder; SCAS‐8‐C, Brief Spence Children's Anxiety Scale‐Child version; SCAS‐8‐P, Brief Spence Children's Anxiety Scale‐Parent version; SCAS‐8‐T, Brief Spence Children's Anxiety Scale‐Teacher version.

In total, parents of 646 children (16.6% of invited) consented and provided parent‐report questionnaire data. Child‐ and teacher‐report questionnaire data were collected for 582 (90.0%) and 565 (87.5%) of these children respectively. Diagnostic outcome data were collected for 463 children (71.7%), and 107 children met diagnostic criteria for an anxiety disorder. Children with and without diagnostic outcome data were similar on gender (female: 52.5%/49.5%), year group (Year 4: 33.0%/37.7%; Year 5: 37.4%/37.7%; Year 6: 29.6%/24.6%), and child anxiety symptoms (SCAS‐8‐P, mean (SD): 6.9 (4.42)/6.8 (4.09)).

### Item selection for candidate measures

#### Item selection for symptom/impact measures

A summary of responses for all items for each reporter are provided in Tables S1‐S3. Participant feedback related to the relevance or clarity of individual items was minimal (fewer than 20 children and fewer than 20 parents reported difficulty understanding or queried the relevance of any single item) so we retained all items for the factor analysis to be inclusive at this initial stage. Exploratory factor analyses including all symptom and impact items identified 6 salient factors for child‐report items, 7 factors for parent‐report items, and 6 factors for teacher‐report items (see Tables S4‐S8).

Findings from backward elimination logistic regression models used to identify items that were the strongest predictors of anxiety disorder status are provided in Table S9. All five items included in the final child‐report model (child‐report candidate measure 1) assessed anxiety symptoms, whereas the final parent‐report model (parent‐report candidate measure 1, version A; seven items) and teacher‐report model (teacher‐report candidate measure 1; three items) included a combination of symptom items and impact items (see Tables 2, 3, 4). Two of the selected parent‐report items were considered to have limited face validity in a very short measure so we replaced them with alternative items to provide an alternative candidate parent‐report symptoms/impact measure (parent‐report candidate measure 1, version B; seven items) (see Appendix 3 for further details).

**TABLE 2:** Child‐report candidate measures.

Candidate measure	N	Number of items	Cut‐off score	AUC (95% CI)	Sensitivity (95% CI)	Specificity (95% CI)
Candidate measure 1 (symptoms/impact)	447	5	≥4 (out of 15)	0.76 (0.71–0.81)	73.3% (63.5%–81.6%)	63.9% (58.6%–68.9%)
*1. I worry that bad things will happen to me*						
*2. I suddenly start to tremble or shake when there is no reason for this*						
*3. I have trouble going to school in the mornings because I feel nervous or afraid*						
*4*. *I worry about things more than other children my age*						
*5*. *I feel afraid if I have to talk in front of my class*						
Candidate measure 2 (2 stage screen)	441	8	≥2 (out of 18) (symptoms) ≥3 (out of 6) (impact)		66.0% (55.8%–75.2%)	67.7% (62.5%–72.7%)
Symptoms						
*1*. *I worry about being away from my parents*						
*2*. *I wake up feeling scared*						
*3*. *I worry about things more than other children my age*						
*4. I have trouble going to school in the mornings because I feel nervous or afraid*						
*5*. *I feel afraid if I have to talk in front of my class*						
*6*. *I try to avoid something because it scares me*						
Impact						
*1. Do fears or worries cause problems for you?*						
*2*. *Would you like some help with fears or worries?*						
Candidate measure 3 (impact only)	449	2	≥3 (out of 6)	0.71 (0.65–0.77)	65.4% (55.2%–74.5%)	66.7% (61.4%–71.6%)
*1. Do fears or worries cause problems for you?*						
*2*. *Would you like some help with fears or worries?*						
SCAS‐8^a^	450	8	Boys: ≥6 (out of 24) Girls: ≥8 (out of 24)	0.72 (0.66–0.78)	67.3% (57.3%–76.3%)	66.2% (61.0%–71.1%)
SDQ‐E^b^	446	5	≥7 out of 10	0.74 (0.68–0.79)	56.0% (45.7%–65.9%)	79.5% (74.8%–83.6%)
RCADS‐25 Anxiety scale^c^	438	15	≥13 (out of 45)	0.70 (0.64–0.76)	68.3% (58.3%–77.2%)	63.8% (58.4%–68.9%)

^a^ Used cut‐off scores provided by Reardon, Spence, et al. (2018).

^b^ Used published cut‐off scores provided by here: https://www.sdqinfo.org.

^c^ Suitable cut‐off scores not available so identified optimal cut‐off score in the current sample.

**TABLE 3:** Parent‐report candidate measures.

	N	Number of items	Cut‐off score	AUC (95% CI)	Sensitivity (95% CI)	Specificity (95% CI)
Candidate measure 1, version A (symptoms and impact)	455	7	≥5 (out of 21)	0.87 (0.83–0.91)	81.6% (72.7%–88.5%)	73.0% (68.1%–77.6%)
*1*. *My child worries they might say or do something stupid in front of other children*						
*2*. *My child worries when they go to bed at night*						
*3*. *My child complains of their heart suddenly starting to beat too quickly for no reason*						
*4*. *My child would feel scared if they had to stay away from home overnight*						
*5*. *My child has to keep checking that they have done things right (like the switch is off, or the door is locked)*						
*6*. *Do fears, worries or anxiety upset or distress your child?*						
*7. Do your child's fears, worries or anxiety make these things difficult for your everyday life in … your relationships with family or friends*						
Candidate measure 1, version B (symptoms and impact)	455	7	≥7 (out of 21)	0.86 (0.82–0.90)	75.0% (65.6%–83.0%)	82.3% (77.9%–86.2%)
*1. My child worries they might say or do something stupid in front of other children*						
*2. My child worries when they go to bed at night*						
*3. My child complains of their heart suddenly starting to beat too quickly for no reason*						
*4. My child would feel scared if they had to stay away from home overnight*						
*5. My child worries that something bad will happen to them*						
*6. Do fears, worries or anxiety upset or distress your child?*						
*7. Do your child's fears, worries or anxiety make things difficult for your family as a whole?*						
Candidate measure 2, version A (two‐stage screen)	450	9	4 ≥ (out of 21) (symptoms) 2 ≥ (out of 6) (impact)		77.0% (67.5%–84.8%)	82.3% (77.9%–86.1%)
Symptoms						
*1. My child has to do certain things in just the right way to stop bad things happening*						
*2*. *My child worries about things more than other children in a similar situation*						
*3*. *My child complains of their heart suddenly starting to beat too quickly for no reason*						
*4*. *My child would feel scared if they had to stay away from home overnight*						
*5. My child can't seem to get bad or silly thoughts out of their head*						
*6*. *My child has to keep checking that they have done things right (like the switch is off, or the door is locked)*						
*7*. *My child is scared if they have to sleep on their own*						
Impact						
*1. Do fears, worries or anxiety upset or distress your child?*						
*2*. *Do your child's fears, worries or anxiety make things difficult for your family as a whole?*						
Candidate measure 2, version B (two‐stage screen)	452	9	4 ≥ (out of 21) (symptoms) 3 ≥ (out of 6) (impact)		75.0% (65.6%–83.0%)	81.6% (77.1%–85.5%)
Symptoms						
*1. My child worries that something awful will happen to someone in our family*						
*2*. *My child finds it hard to stop worrying*						
*3*. *My child complains of their heart suddenly starting to beat too quickly for no reason*						
*4*. *My child would feel scared if they had to stay away from home overnight*						
*5. My child can't seem to get bad or silly thoughts out of their head*						
*6*. *My child worries they might say or do something stupid in front of other children*						
7. *My child is scared if they have to sleep on their own*						
Impact						
*1. Do fears, worries or anxiety upset or distress your child?*						
*2*. *Do your child's fears, worries or anxiety make things difficult for your family as a whole?*						
Candidate measure 3 (impact only)	460	2	≥3 (out of 6)	0.85 (0.80–0.89)	76.6% (67.5%–84.3%)	79.6% (75.0%–83.7%)
*1. Do fears, worries or anxiety upset or distress your child?*						
*2*. *Do your child's fears, worries or anxiety make things difficult for your family as a whole?*						
SCAS‐8^a^	455	8	≥8 (out of 24)	0.82 (0.77–0.87)	76.7% (67.3%–84.5%)	73.6% (68.6%–78.1%)
SDQ‐E^b^	456	5	≥5 out of 10	0.81 (0.76–0.86)	75.2% (65.9%–83.1%)	72.9% (68.0%–77.5%)
RCADS‐25 Anxiety scale^c^	443	15	≥11 (out of 45)	0.84 (0.80–0.89)	77.7% (68.4%–85.3%)	75.9% (71.0%–80.3%)

^a^ Used cut‐off scores provided by Reardon, Spence, et al. (2018).

^b^ Used published cut‐off scores provided by here: https://www.sdqinfo.org

^c^ Suitable cut‐off scores not available so identified optimal cut‐off score in the current sample.

**TABLE 4:** Summary of teacher‐report candidate measures.

	N	Number of items	Cut‐off score	AUC (95% CI)	Sensitivity (95% CI)	Specificity (95% CI)
Candidate measure 1 (symptoms/impact)	408	3	≥2 (out of 9)	0.74 (0.69–0.80)	73.5% (63.6%–81.9%)	62.6% (56.9%–68.0%)
*1*. *Talks in front of other children in the class, when appropriate to do so*						
*2. Do fears, worries or anxiety cause problems for this child?*						
3. *Do this child's fears, worries or anxiety make things difficult for you or the class as a whole?*						
Candidate measure 2 (2 stage screen)	389	7	≥2 (out of 15) (symptoms) ≥1 (out of 6) (impact)		75.8% (65.9%–84.0%)	62.6% (56.8%–68.1%)
Symptoms						
*1. Can't seem to get bad or silly thoughts out of their head*						
2. *Worries when they think they have done poorly at something*						
3. *Talks in front of teachers, when appropriate to do so*						
*4. Worries about things*						
*5. Talks in front of other children in the class, when appropriate to do so*						
Impact						
*1. Do fears, worries or anxiety upset or distress this child?*						
*2. Do fears, worries or anxiety stop this child from doing things?*						
Candidate measure 3 (impact only)	407	2	≥1 (out of 6)	0.75 (0.70–0.81)	77.8% (68.3%–85.5%)	61.4% (55.7%–66.8%)
*1. Do fears, worries or anxiety upset or distress this child?*						
*2. Do fears, worries or anxiety stop this child from doing things?*						
SCAS‐8^a^	381	8	Boys: ≥4 (out of 24) Girls: ≥5 (out of 24)	0.69 (0.63–0.75)	56.4% (45.8%–66.6%)	73.9% (68.4%–78.9%)
SDQ‐E^b^	384	5	≥6 out of 10	0.69 (0.63–0.76)	29.0% (20.1%–39.4%)	92.8% (89.2%–95.5%)
RCADS‐25 Anxiety scale^c^	372	15	≥3 (out of 39)	0.66 (0.59–0.72)	70.3% (59.8%–79.5%)	51.6% (45.6%–57.6%)

^a^ Used cut‐off scores provided by Reardon, Spence, et al. (2018).

^b^ Used published cut‐off scores provided by here: https://www.sdqinfo.org.

^c^ Suitable cut‐off scores not available so identified optimal cut‐off score in the current sample.

#### Item selection for separate symptom and impact measures

##### Symptom item selection

As detailed in Tables S10‐S14, exploratory factor analyses including symptom items only identified 5 factors for child‐report items, 6 factors for parent‐report items, and 5 factors for teacher‐report items. Following the same backward elimination multivariable logistic regression approach as before, including items with the highest loading on each symptom factor, followed by consideration of all removed symptom items, we identified 6 child‐report symptom items (child‐report candidate measure 2), 7 parent‐report symptom items (parent‐report candidate measure 2, version A), and 5 teacher‐report symptom items (teacher‐report candidate measure 2) that were the strongest predictors of anxiety disorder status (see Table S15). Notably, there was some overlap in items selected for candidate symptom measures and those previously selected from the whole pool of symptom and impact items (see Tables 2, 3, 4). We replaced three of the selected parent‐report symptom items (see Appendix 3) to provide an alternative candidate parent‐report symptoms measure (parent‐report candidate measure 2, version B).

##### Impact item selection

To reduce the impact item pool, we omitted items related to impairment in specific domains and the duration of symptoms that may have limited face validity in a short measure, leaving 5 child‐report, 6 parent‐report and 5 teacher‐report impact items. Findings from backward elimination logistic regression analyses using these impact items are detailed in Table S16, and identified 2 child‐report, 2 parent‐report, and 2 teacher‐report impact items for inclusion in candidate ‘two‐stage’ and ‘impact‐only’ measures (see Tables 2, 3, 4).

### Evaluation of candidate measures

Tables 2 to 4 detail for each reporter the AUC, and sensitivity and specificity for optimal cut‐off scores for each candidate measure, together with corresponding values for the SCAS‐8, RCADS‐25‐Anxiety Scale and SDQ‐E. None of the evaluated measures achieved the optimal sensitivity and specificity of 85%, but several parent‐report measures achieved ≥75% sensitivity and specificity. Indeed, alternative candidate parent‐report measures performed similarly, with sensitivity/specificity values ≥75% for one combined symptoms/impact measure (7 items: sensitivity/specificity: 75% [95% CI 66%–83%]/82% [95% CI 78%–86%]), both two‐stage screens (9 items: sensitivity/specificity 77% [95% CI 68%–85%]/82% [95% CI 78%–86%]; 75% [95% CI 66%–83%]/82% [95% CI 77%–86%]) and the impact‐only measure (2 items: sensitivity/specificity: 77% [95% CI 68%–84%]/80% [95% CI 75%–84%]). The parent‐report RCADS‐25‐Anxiety Scale also achieved ≥75% sensitivity and specificity (78% [95% CI 68%–85%]/76% [95% CI 71%–80%]), and the parent‐report SCAS‐8 and SDQ‐E both achieved >73% sensitivity/specificity (SCAS‐8: 77% [95% CI 67%–85%]/74% [95% CI 69%–78%]; SDQ‐E: 75% [95% CI 66%–83%]/73% [95% CI 68%–78%]).

Subgroup analyses indicated the candidate parent‐report measures that achieved acceptable sensitivity/specificity overall, performed similarly across gender, age and ethnicity subgroups (see Table S17). Although notably, only a subset (27%–33%) of the ‘false positives’ on each of these parent‐report measures were assigned a subclinical anxiety disorder diagnosis on the ADIS‐C/P.

None of the candidate child‐report or teacher‐report measures or comparison child/teacher‐report questionnaires achieved sensitivity and specificity levels of ≥75%. Among the child‐report measures, ‘candidate measure 1’ including 5 symptom items selected from the whole symptom/impact pool achieved the best sensitivity/specificity (73%/64%). Each of the new candidate teacher‐report measures performed similarly (sensitivity/specificity: 74%/63%; 76%/63%; 78%/61%), while none of the comparison teacher‐report measures (SCAS‐8, RCADS‐25‐Anxiety Scale, SDQ‐E) achieved >60% for both sensitivity and specificity.

As neither child‐ nor teacher‐report measures achieved an acceptable level of sensitivity and specificity, if either one or both was combined with a parent‐report measure, any improvement to sensitivity would inevitably have been at the expense of reduced specificity so these analyses were not conducted.

## DISCUSSION

This study aimed to develop an assessment tool that can be used to identify children with anxiety disorders in community settings. We found that alternative candidate parent‐report questionnaires consisting of between two and nine items were able to identify children with/without anxiety disorders with a similar level of accuracy (≥75% sensitivity and specificity). The parent‐report measure that consists of only 2 ‘impact’ items (*Do fears, worries or anxiety upset or distress your child? Do your child's fears, worries or anxiety make things difficult for your family as a whole?*) achieved 77% sensitivity and 80% specificity. Alternative parent‐report measures that include these 2 ‘impact’ items together with an additional five to seven items that assess the frequency of specific anxiety symptoms achieved similar levels of sensitivity/specificity (75%/82%; 77%/82%; 75%/82% respectively). Given the two‐item parent‐report measure brings the advantage of brevity, this questionnaire appears optimal in the context of screening where minimising time burden is a priority. It is nevertheless important to acknowledge that none of the parent‐report measures we developed or existing brief parent‐report measures (SCAS‐8; RCADS‐25‐Anxiety Scale; SDQ‐E) achieved the optimal sensitivity and specificity of ≥85%. In the current sample, almost one‐quarter of children with diagnosable anxiety disorders scored below the optimal cut‐off on the two‐item parent‐report measure (false negatives) and approximately one‐fifth of children who did not meet diagnostic criteria for an anxiety disorder scored above the cut‐off (false positives). Therefore, prior to using the two‐item parent‐report measure for screening, careful consideration of ways to minimise negative consequences from these relatively sizeable numbers of false negatives and false positives will be critical (Williamson et al., 2021).

None of the child‐ or teacher‐report candidate questionnaires we developed or existing brief child/teacher‐report questionnaires (SCAS‐8, RCADS‐25‐Anxiety Scale; SDQ‐E) achieved a sufficient level of accuracy (all <75% sensitivity or specificity) for us to consider them as suitable for screening purposes. These findings are in line with previous studies that indicate parent‐report should be prioritised in the context of identifying anxiety disorders among primary‐school aged children. For example, the parent‐report SCAS (full length and eight item versions) is better than either the child‐ or teacher‐report SCAS/SCAS‐8 at discriminating a clinic‐referred sample of children with anxiety disorders from a community sample (Reardon, Spence, et al., 2018), and parent‐reported interference related to anxiety symptoms (CAIS‐P) is a better predictor of recovery from anxiety disorders in clinical samples than child‐reported interference (CAIS‐C) (Evans et al., 2017). However, it is important to note that our finding that parent‐report questionnaires were the strongest predictors of diagnostic status could relate to the dominant role of parent‐report in the ADIS‐C/P among primary‐school aged children (Grills & Ollendick, 2003). Equally, patterns across different reporters vary among different age groups, and our finding that parent‐report was optimal for identifying anxiety disorders among primary‐school aged children may not apply among adolescents.

Our findings highlight the benefit of assessing impact associated with anxiety symptoms to improve identification of child anxiety disorders. Indeed, the finding that the two‐item parent‐report measure that *only* assessed distress and impairment achieved a similar level of accuracy to longer parent‐report measures that assessed *both* symptoms *and* distress and impairment, suggests assessment of impact may be more important than assessment of symptoms in the context of screening in community settings. Measures of impact have been shown to be advantageous compared to symptom measures in the context of identifying recovery from anxiety disorders in clinical samples (Evans et al., 2017), and discriminating community samples from clinic‐referred samples with anxiety and depressive disorders (Radez et al., 2021), and referrals to child and adolescent mental health services more broadly (Goodman, 1999). Importantly, our study extends these previous findings to the context of identifying anxiety disorders within a community sample and indicate that as few as two items designed to assess impact associated with anxiety symptoms are able to identify children (aged 8–11) with/without anxiety disorders with reasonable accuracy. Future studies need to consider whether using very short measures assessing impact associated with anxiety symptoms could be a useful approach to screening for anxiety disorders in younger children (<8 years) and adolescents (>11 years) in school or primary care settings, and whether a similar approach could be used to screen for other specific mental health disorders in children and adolescents.

### Implications

This two‐item parent‐report impact measure has the potential to be used in community settings as a tool to improve identification of anxiety disorders among children aged 8–11 years. Consisting of only two items means that this measure is very quick and easy to complete and score, which are particular advantages in the context of screening in schools and other settings such as primary care. The brevity of this measure also means it lends itself to use alongside similar tools designed to improve identification of other specific mental health disorders. However, screening is only recommended where the benefits of screening outweigh any potential harms (e.g. from false negatives/false positives) and where effective intervention is available for those identified through screening (UK National Screening Committee, 2022). Potential negative consequences of false positives and false negatives could be minimised by: (a) working with parents and school staff to develop procedures for sharing screening outcomes with families to help ensure the possibility of inaccurate screening outcomes are clear, and (b) as well as actively offering an intervention to children who screen positive, also making support and intervention available for other families who request it (Williamson et al., 2022). Prior to using the two‐item parent‐report measure in primary care settings, it will be important to ensure support and intervention will be available to those children who are identified from screening, and procedures for sharing screening outcomes with families in this context are developed together with families and practitioners.

### Strengths and limitations

A particular strength of this study is that we used a standardised diagnostic assessment (ADIS‐C/P) to determine whether children in a large community sample met diagnostic criteria for an anxiety disorder. This approach allowed us to evaluate the capacity of alternative candidate measures to discriminate between children with and without anxiety disorders *within* a community sample. Indeed, to the best of our knowledge, this is the first study to provide data on the capacity of short child, parent and teacher‐report child anxiety measures to identify primary‐school aged children with/without anxiety disorders. Moreover, socio‐demographic characteristics of our sample were broadly reflective of the UK general population (e.g., 64% owned their own house compared to 63% of households in England (Ministry of Housing, 2020), although White British children were over‐represented (82% compared to 74% in the population in England and Wales (Office for National Statistics, 2021)).

There are several limitations with this study. We developed and evaluated candidate questionnaire measures using a single sample so ideally future research will evaluate these measures in an independent community sample, with a sufficient sample size to explore subgroups meaningfully. Parental written consent was required for study participation and the procedures (including a full diagnostic assessment) were time consuming for parents; as such, the response rate was relatively low (16.6% of invited children). The proportion of children who met diagnostic criteria for an anxiety disorder (23%) is notably higher than estimated prevalence rates, indicating there was a likely participation bias where parents who were concerned about their child's anxiety were most likely to participate. It is therefore possible that levels of accuracy for candidate measures could differ in other samples, where for example, there are larger proportions of children without anxiety disorders or subclinical anxiety problems. Indeed, it is possible that this study underestimates the ability of the brief questionnaires to accurately identify children *without* anxiety disorders (i.e. specificity).

## CONCLUSIONS

Brief parent‐report child anxiety questionnaires can discriminate between children with and without anxiety disorders within a community sample with an acceptable level of accuracy. Our findings highlight the benefit of assessing impact associated anxiety symptoms in the context of screening, with just two parent‐report items that assess impact achieving >75% sensitivity and specificity. This two‐item measure offers an efficient tool for use in community settings to identify children who may benefit from support and intervention for anxiety problems.

## AUTHOR CONTRIBUTIONS

**Tessa Reardon**: Conceptualization; Data curation; Investigation; Methodology; Project administration; Supervision; Visualization; Writing – original draft; Writing – review & editing. **Obioha C. Ukoumunne**: Conceptualization; Data curation; Formal analysis; Funding acquisition; Methodology; Visualization; Writing – review & editing. **Susan Ball**: Conceptualization; Data curation; Formal analysis; Methodology; Writing – review & editing. **Paul Brown**: Conceptualization; Funding acquisition; Methodology; Writing – review & editing. **Tamsin Ford**: Conceptualization; Funding acquisition; Methodology; Writing – review & editing. **Alastair Gray**: Conceptualization; Funding acquisition; Methodology; Writing – review & editing. **Claire Hill**: Conceptualization; Funding acquisition; Methodology; Writing – review & editing. **Bec Jasper**: Conceptualization; Funding acquisition; Methodology; Writing – review & editing. **Michael Larkin**: Conceptualization; Funding acquisition; Methodology; Writing – review & editing. **Ian Macdonald**: Conceptualization; Funding acquisition; Methodology; Writing – review & editing. **Fran Morgan**: Conceptualization; Funding acquisition; Methodology; Writing – review & editing. **Michelle Sancho**: Conceptualization; Funding acquisition; Methodology; Writing – review & editing. **Falko F. Sniehotta**: Conceptualization; Funding acquisition; Methodology; Writing – review & editing. **Susan H. Spence**: Conceptualization; Funding acquisition; Methodology; Writing – review & editing. **Jason Stainer**: Conceptualization; Funding acquisition; Methodology; Writing – review & editing. **Paul Stallard**: Conceptualization; Funding acquisition; Methodology; Writing – review & editing. **Mara Violato**: Conceptualization; Funding acquisition; Methodology; Writing – review & editing. **Cathy Creswell**: Conceptualization; Funding acquisition; Investigation; Methodology; Project administration; Supervision; Writing – review & editing.

## CONFLICT OF INTEREST STATEMENT

The authors have declared no competing or potential conflicts of interest.

## ETHICAL CONSIDERATIONS

The study was approved by the University of Oxford Medical Sciences Interdivisional Research Ethics Committee (Reference: R64592). Parents provided written consent and children provided written assent.

## Supporting information

Supporting Information S1

## Data Availability

The data that support the findings of this study are available from the corresponding author upon reasonable request.
