# Supplementary material for: Development of a brief assessment tool to identify children with probable anxiety disorders
Source: JCPP Adv. 2024 Aug 17;5(2):e12265. doi: 10.1002/jcv2.12265 (PMC12159304; doi:10.1002/jcv2.12265)
Supplement: Supplementary file 1 — Supporting Information S1 [file JCV2-5-e12265-s001.docx]

Appendix 1

Sample size

Our original recruitment target was 770 children. We anticipated that to achieve this we would need to recruit approximately 22 schools (based on an estimated response rate of 20% and 180 eligible children per school). Based on an estimated prevalence rate of 6.5% (Polanczyk et al., 2015), we anticipated 50 out of 770 children would have an anxiety disorder diagnosis on the basis of the ADIS-C/P. Although there are no clearly defined and agreed levels of sensitivity and specificity required for school-based screening, a minimum of 75-85% for both is often recommended (Glover & Albers, 2007). We therefore considered sensitivity and specificity of ≥85% as optimal, and ≥75% as acceptable. Fifty children with an anxiety disorder is sufficient to estimate a sensitivity of 85% with a standard error of 5 percentage points and 720 children without an anxiety disorder is enough to estimate a specificity of 85% with a standard error of 1.3 percentage points.

The study required us to use backward elimination logistic regression to identify the best items (predictors) out of a candidate set in the model for predicting the binary outcome anxiety disorder status. We used published criteria (Riley, et al 2020) to ensure that our target sample size (770 participants) is large enough to do this given the following assumptions: (a) we anticipated specifying between 5 and 10 items as predictors in the models; (b) the prevalence of anxiety disorder is 6.5%.

The target sample size is large enough to estimate the prevalence of anxiety disorder with a margin of error of 2.0% based on the upper bound of the 95% confidence interval; this is smaller than the 5% margin of error that has been recommended (Riley et al, 2020).

The sample size is also large enough to keep the mean absolute error in the predicted probability of anxiety disorder across individuals to 0.018 in backward elimination logistic regression models that include 5 candidate predictors and to 0.026 in models that include 10 candidate predictors; it is recommended that the mean absolute prediction error is no greater than 0.05 (Riley et al, 2020).

To ensure that only a small amount of shrinkage (10%) (Riley et al., 2020) is required to allow for model overfitting, up to 5 candidate predictors may be specified if the pseudo (Cox-Snell) R squared measure of model fit is 0.056 and up to 10 candidate predictors may be specified if the pseudo R squared statistic is 0.11.

Finally, the expected optimism in the apparent pseudo R squared statistic is 0.0175 if 5 candidate predictors are specified and 0.035 if 10 candidate predictors are specified; it has been recommended that the expected optimism should be no more than 0.05 (Riley et al., 2020).

**References**

Polanczyk, G., Salum, G., Sugaya, L, Caye, A., & Rohde, L. (2015). Annual research review: a meta-analysis of the worldwide prevalence of mental disorders in children and adolescents. *Journal of Child Psychology and Psychiatry*, 56(3), 345–365.

Glover, T., & Albers, C. (2007). Considerations for evaluating universal screening assessments. *Journal of School Psychology*, *45*, 117–135.

Riley, R.D., Ensor, J., Snell, K., Harrell, F.E., Martin, G.P.,…van Smeden, M. (2020). Calculating the sample size required for developing a clinical prediction model. *BMJ,* 368, m441

Appendix 2

Measures

*Child anxiety questionnaire items (child-, parent- and teacher- report versions)*

A full list of the child anxiety items completed by children, parents and teachers is available here: <https://osf.io/24dcz>.

*Symptom items*

The symptom items were primarily items from the Spence Children’s Anxiety Scale (SCAS) and the Revised Children’s Anxiety and Depression Scale (RCADS)-Anxiety Scale. The SCAS and the RCADS-Anxiety Scale (which is partially derived from the SCAS) are designed to assess symptoms of anxiety disorders. Both have been widely evaluated in community and clinical populations, with good evidence to support their strong psychometric properties (see <https://www.scaswebsite.com/> and <https://rcads.ucla.edu/> for further information and publication lists).

The only SCAS items that we omitted for all reporters were the items that assess the frequency of specific fears (e.g. fears of dogs, spiders) as these items have limited capacity to discriminate between children with and without specific phobias (Reardon et al., 2019) and so are unlikely to be suitable for inclusion in a brief screening tool. Although Obsessive Compulsive Disorder is not classified as an anxiety disorder in the DSM-5, we included the SCAS items that assess obsessive and compulsive behaviours because some of these item scores correlate highly with SCAS total scores (Reardon et al., 2018) so may capture symptoms that are common across anxiety disorders and therefore could be suitable for inclusion in a brief questionnaire.

In addition to the SCAS items, we included the 8 items from the RCADS-Anxiety Scale that do not appear on the SCAS and additional symptom items developed for this study. New symptom items included items to assess selective mutism (e.g., *I talk to other children in my class*), additional symptoms of social anxiety (e.g., *I feel scared when I meet new people*) and generalised anxiety (e.g., *I feel nervous, I worry about things more than other children my age*), and items to assess the presence of any specific fear (e.g., *I try to avoid something because it scares me*).

We used child- and parent-report versions of the SCAS/RCADS/additional symptom items, with minor amendments made to the item wording to make them suitable for teachers (e.g., *I worry about things* amended to *worries about things*). For the teacher-report version, we omitted items that teachers were unlikely to observe (e.g., items related to sleep).

*Impact items*

Items to assess impact associated with anxiety symptoms were developed for this study, drawing on similar items from the Strengths and Difficulties Questionnaire impact supplement (see <https://www.sdqinfo.org/> for further information), and two questionnaire measures that have been shown to be reliable and valid measures of interference caused by anxiety symptoms in clinical populations (Child Anxiety Impact Scale, Langley et al., 2014; and Child Anxiety Life Interference Scale, Lyneham et al., 2013).

*Anxiety Disorder Interview Schedule Child Version: Child and Parent Interviews (ADIS-C/P)*

In cases where only one interview (child or parent), we followed our pre-specified protocol (<https://osf.io/y7na6>): 1) if the child met diagnostic criteria for an anxiety disorder with a CSR 4-8 on the basis of the completed interview, the child was classified as ‘anxiety diagnosis’ and included in the main analyses that used diagnostic outcomes. This was on the basis that whatever the outcome of the missing interview, the overall classification would not have changed; 2) if the child did not meet diagnostic criteria for an anxiety disorder with a CSR 4-8, the diagnostic outcome was considered as missing and the child was not included in the main analysis because if the child had met diagnostic criteria on the basis of the missing interview, the overall classification would have changed. In cases where neither child nor parent interview were completed, the diagnostic outcome was treated as missing and the child was not included in the main analysis.

References

Ebesutani, C., Korathu-Larson, P., Nakamura, B. J., Higa-McMillan, C., & Chorpita, B. (2017). The Revised Child Anxiety and Depression Scale 25–Parent Version: Scale Development and Validation in a School-Based and Clinical Sample. *Assessment*, *24*(6), 712–728. <https://doi.org/10.1177/1073191115627012>

Ebesutani, C., Reise, S. P., Chorpita, B. F., Ale, C., Regan, J., Young, J., Higa-McMillan, C., & Weisz, J. R. (2012). The Revised Child Anxiety and Depression Scale-Short Version: Scale reduction via exploratory bifactor modeling of the broad anxiety factor. *Psychological Assessment*, *24*(4), 833–845. <https://doi.org/10.1037/a0027283>

Langley, A. K., Falk, A., Peris, T., Wiley, J. F., Kendall, P. C., Ginsburg, G., Birmaher, B., March, J., Albano, A. M., & Piacentini, J. (2014). The Child Anxiety Impact Scale: Examining Parent- and Child-Reported Impairment in Child Anxiety Disorders. *Journal of Clinical Child and Adolescent Psychology*, *43*(4), 579–591. <https://doi.org/10.1080/15374416.2013.817311>

Lyneham, H. J., Sburlati, E. S., Abbott, M. J., Rapee, R. M., Hudson, J. L., Tolin, D. F., & Carlson, S. E. (2013). Psychometric properties of the child anxiety life interference scale (CALIS). *Journal of anxiety disorders*, *27*(7), 711-719.

Reardon, T., Creswell, C., Lester, K. J., Arendt, K., Blatter-Meunier, J., Bögels, S. M., … &Eley, T. C. (2019). The utility of the SCAS-C/P to detect specific anxiety disorders among clinically anxious children. *Psychological Assessment*, *31*(8), 1006-1018. <https://doi.org/10.1037/pas0000700>

Reardon, T., Spence, S. H., Hesse, J., Sahkir, A., & Creswell, C. (2018). Identifying children with anxiety disorders using brief versions of the Spence Children’s Anxiety Scale for children, parents, and teachers. *Psychological Assessment*, *30*(10), 1342–1355. <https://doi.org/10.1037/pas0000570>

Appendix 3

Item selection for candidate measures

*Item selection for symptom/impact measures*

One of the selected parent-report items related to compulsive checking behaviour (*My child has to keep checking that they have done things right)*, and another to one specific area of functional impairment (impact on parent’s relationships with family or friends). These items were considered to have limited face validity in a very short measure so we replaced them with alternative items related to generalised anxiety (*My child worries that something bad will happen to them*) and more general impact on family life (*Do your child's fears, worries or anxiety make things difficult for your family as a whole?*) to provide an alternative candidate parent-report symptoms/impact measure (parent-report candidate measure 1, version B; 7 items).

*Item selection for separate symptom and impact measures*

*Symptom item selection*

Two of the selected parent-report symptom items related to obsessive/compulsive behaviours (*My child has to keep checking that they have done things right; My child has to do certain things in just the right way to stop bad things happening)* may have limited face validity in a very short measure, and one of the selected parent-report items was identified by a small number of parents as difficult to answer (*My child worries about things more than other children in a similar situation*). We therefore replaced these items with alternative items (*My child worries that something awful will happen to someone in our family*; *My child finds it hard to stop worrying*; *My child worries they might say or do something stupid in front of other children*) to provide an alternative candidate parent-report symptoms measure (parent-report candidate measure 2, version B).

Table S1 Summary of responses for child-report symptom and impact items (66 items, plus 6 positive filler items from the SCAS)

| **Item label** | **Item** |  | **N** |  | **Response percentage** | | | |
| --- | --- | --- | --- | --- | --- | --- | --- | --- |
|  |  |  |  |  | **0** | **1** | **2** | **3** |
|  | **Symptom items^a^** |  |  |  |  |  |  |  |
| worry_c | 1. I worry about things |  | 582 |  | 6.2 | 63.2 | 23.9 | 6.7 |
| stom_c | 2. When I have a problem, I get a funny feeling in my stomach |  | 581 |  | 41.7 | 39.8 | 11.4 | 7.2 |
| afraid_c | 3. I feel afraid |  | 580 |  | 19.5 | 64.0 | 13.3 | 3.3 |
| alone_c | 4. I would feel afraid of being on my own at home |  | 576 |  | 41.8 | 29.5 | 10.2 | 18.4 |
| test_c | 5. I feel scared when I have to take a test |  | 582 |  | 43.6 | 34.4 | 11.5 | 10.5 |
| nerves_c | 6. I feel nervous |  | 582 |  | 13.4 | 60.8 | 20.4 | 5.3 |
| toilet_c | 7. I feel afraid if I have to use public toilets or bathrooms |  | 579 |  | 67.2 | 19.5 | 7.1 | 6.2 |
| awayp_c | 8. I worry about being away from my parents |  | 581 |  | 26.5 | 43.4 | 12.9 | 17.2 |
| fool_c | 9. I feel afraid that I will make a fool of myself in front of people |  | 580 |  | 36.2 | 37.2 | 16.4 | 10.2 |
| work_c | 10. I worry that I will do badly at my school work |  | 582 |  | 33.2 | 44.0 | 14.4 | 8.4 |
| pop_c | 11. I am popular amongst other kids my own age^b, c^ |  | 579 |  | 10.4 | 20.7 | 41.5 | 27.5 |
| talkch_c | 12. I talk in front of other children in my class^c^ |  | 580 |  | 23.1 | 27.2 | 35.3 | 14.3 |
| awffam_c | 13. I worry that something awful will happen to someone in my family |  | 582 |  | 25.6 | 41.2 | 16.8 | 16.3 |
| breath_c | 14. I suddenly feel as if I can't breathe when there is no reason for this |  | 582 |  | 71.3 | 17.5 | 7.6 | 3.6 |
| check_c | 15. I have to keep checking that I have done things right (like the switch is off, or the door is locked) |  | 581 |  | 37.5 | 36.1 | 13.6 | 12.7 |
| sleep_c | 16. I feel scared if I have to sleep on my own |  | 582 |  | 61.3 | 25.3 | 6.9 | 6.5 |
| school_c | 17. I have trouble going to school in the mornings because I feel nervous or afraid |  | 582 |  | 67.4 | 23.2 | 5.7 | 3.8 |
| sports_c | 18. I am good at sports^b, c^ |  | 581 |  | 36.5 | 27.7 | 27.5 | 8.3 |
| silly_c | 19. I can't seem to get bad or silly thoughts out of my head |  | 574 |  | 27.7 | 41.8 | 18.1 | 12.4 |
| heart_c | 20. When I have a problem, my heart beats really fast |  | 581 |  | 38.7 | 37.0 | 14.6 | 9.6 |
| trembl_c | 21. I suddenly start to tremble or shake when there is no reason for this |  | 582 |  | 69.2 | 21.3 | 6.4 | 3.1 |
| bad_c | 22. I worry that something bad will happen to me |  | 581 |  | 32.0 | 43.5 | 15.0 | 9.5 |
| shaky_c | 23. When I have a problem, I feel shaky |  | 582 |  | 47.8 | 34.4 | 9.6 | 8.2 |
| good_c | 24. I am a good person^b, c^ |  | 580 |  | 48.3 | 38.1 | 12.4 | 1.2 |
| specl_c | 25. I have to think of special thoughts to stop bad things from happening (like numbers or words) |  | 580 |  | 54.7 | 27.6 | 11.7 | 6.0 |
| stupid_c | 26. I worry I might say or do something stupid in front of other children |  | 582 |  | 36.4 | 36.8 | 16.0 | 10.8 |
| travl_c | 27. I feel scared if I have to travel in the car, or on a bus or a train |  | 582 |  | 70.4 | 18.4 | 6.2 | 5.0 |
| othink_c | 28. I worry what other people think of me |  | 580 |  | 30.9 | 39.1 | 17.1 | 12.9 |
| crowd_c | 29. I am afraid of being in crowded places (like shopping centres, the movies, buses, busy playgrounds) |  | 581 |  | 54.0 | 27.2 | 9.6 | 9.1 |
| happy_c | 30. I feel happy^b, c^ |  | 582 |  | 25.8 | 54.3 | 18.7 | 1.2 |
| scared_c | 31. All of a sudden I feel really scared for no reason at all |  | 579 |  | 60.8 | 27.6 | 7.3 | 4.3 |
| dizzy_c | 32. I suddenly become dizzy or faint when there is no reason for this |  | 582 |  | 79.0 | 15.3 | 3.3 | 2.4 |
| front_c | 33. I feel afraid if I have to talk in front of my class |  | 582 |  | 41.2 | 36.9 | 11.5 | 10.3 |
| wakeup_c | 34. I wake up feeling scared |  | 581 |  | 70.1 | 22.2 | 4.8 | 2.9 |
| hrt_nr_c | 35. My heart suddenly starts to beat too quickly for no reason |  | 580 |  | 75.5 | 17.6 | 4.1 | 2.8 |
| wscare_c | 36. I worry that I will suddenly get a scared feeling when there is nothing to be afraid of |  | 582 |  | 61.7 | 25.8 | 7.4 | 5.2 |
| like_c | 37. I like myself^b, c^ |  | 579 |  | 44.9 | 29.0 | 20.9 | 5.2 |
| small_c | 38. I am afraid of being in small closed places, like tunnels or small rooms. |  | 580 |  | 49.5 | 29.0 | 9.3 | 12.2 |
| repeat_c | 39. I have to do some things over and over again (like washing my hands, cleaning or putting things in a certain order) |  | 582 |  | 41.8 | 32.5 | 12.2 | 13.6 |
| bother_c | 40. I get bothered by bad or silly thoughts or pictures in my mind |  | 579 |  | 36.3 | 37.3 | 16.6 | 9.8 |
| stpbad_c | 41. I have to do some things in just the right way to stop bad things happening |  | 579 |  | 50.8 | 33.5 | 10.0 | 5.7 |
| proud_c | 42. I am proud of my school work^b, c^ |  | 582 |  | 27.8 | 37.5 | 28.2 | 6.5 |
| awayh_c | 43. I would feel scared if I had to stay away from home overnight |  | 582 |  | 50.7 | 30.4 | 8.1 | 10.8 |
| poorly_c | 44. I worry when I think I have done poorly at something |  | 581 |  | 25.6 | 49.7 | 15.5 | 9.1 |
| angry_c | 45. I feel worried when I think someone is angry with me |  | 582 |  | 17.9 | 42.4 | 24.2 | 15.5 |
| flish_c | 46. I worry I might look foolish |  | 582 |  | 39.7 | 37.6 | 15.8 | 6.9 |
| talkt_c | 47. I talk in front of my teachers^c, f^ |  | 576 |  | 29.2 | 24.8 | 32.5 | 13.5 |
| awfslf_c | 48. I worry that bad things will happen to me |  | 582 |  | 34.9 | 39.2 | 17.4 | 8.6 |
| mistak_c | 49. I worry about making mistakes |  | 582 |  | 27.8 | 43.1 | 19.2 | 9.8 |
| happen_c | 50. I worry about what is going to happen |  | 582 |  | 30.2 | 42.6 | 18.9 | 8.2 |
| death_c | 51. I think about death |  | 579 |  | 40.8 | 34.7 | 13.8 | 10.7 |
| wbed_c | 52. I worry when I go to bed at night |  | 580 |  | 52.2 | 29.3 | 10.5 | 7.9 |
| qsttch_c | 53. When I need to, I ask my teacher questions ^c^ |  | 580 |  | 30.5 | 25.0 | 33.4 | 11.0 |
| stpwry_c | 54. I find it hard to stop worrying |  | 581 |  | 33.6 | 34.9 | 17.7 | 13.8 |
| newppl_c | 55. I feel scared when I meet new people |  | 581 |  | 35.8 | 40.6 | 14.6 | 9.0 |
| embras_c | 56. I am afraid I'll do something embarrassing |  | 581 |  | 33.7 | 36.5 | 19.8 | 10.0 |
| w_age_c | 57. I worry about things more than other children my age^f^ |  | 575 |  | 43.0 | 35.1 | 12.9 | 9.0 |
| avoid_c | 58. I try to avoid something because it scares me |  | 580 |  | 28.4 | 41.7 | 17.6 | 12.2 |
| else_c | 59. I am afraid of something else (e.g. dogs, the dark, going to the doctor or dentist, being sick) |  | 581 |  | 28.1 | 37.7 | 19.3 | 15.0 |
|  | **Impact items^d^** |  |  |  |  |  |  |  |
| prob_c | 60. Do fears or worries cause problems for you? |  | 581 |  | 25.0 | 49.6 | 18.4 | 7.1 |
| howlong_c | 61. If yes, how long have you had problems with fears or worries?^e^ |  | 580 |  | 49.1 | 11.6 | 7.4 | 31.9 |
| upset_c | 62. Do fears or worries upset you? |  | 581 |  | 18.6 | 45.3 | 25.1 | 11.0 |
| stop_c | 63. Do fears or worries stop you from doing things? |  | 581 |  | 37.3 | 42.2 | 16.2 | 4.3 |
| cfrds_c | 64. Do fears or worries mess things up for you in these areas? a. making friends or keeping friends |  | 581 |  | 52.2 | 33.2 | 9.8 | 4.8 |
| cfun_c | 65. Do fears or worries mess things up for you in these areas? b. having fun |  | 580 |  | 55.0 | 27.6 | 8.8 | 8.6 |
| cplaces_c | 66. Do fears or worries mess things up for you in these areas? c. going places |  | 581 |  | 51.1 | 33.0 | 10.5 | 5.3 |
| cwork_c | 67. Do fears or worries mess things up for you in these areas? d. doing schoolwork or homework |  | 581 |  | 62.3 | 20.0 | 10.0 | 7.7 |
| calongf_c | 68. Do fears or worries mess things up for you in these areas? e. getting along with family |  | 579 |  | 62.9 | 20.9 | 9.8 | 6.4 |
| csleep_c | 69. Do fears or worries mess things up for you in these areas?f. sleeping |  | 581 |  | 44.8 | 27.2 | 15.8 | 12.2 |
| clife_c | 70. Do fears or worries mess things up for you in these areas? g. life in general |  | 580 |  | 43.4 | 32.6 | 14.7 | 9.3 |
| fam_c | 71. Do your fears or worries make things difficult for people around you (e.g. family, friends, teachers)? |  | 578 |  | 52.1 | 36.2 | 8.8 | 2.9 |
| chelp_c | 72. Would you like some help with fears or worries? |  | 578 |  | 33.9 | 31.7 | 18.5 | 15.9 |

a Symptom item response options: Never (0), Sometimes (1), Often (2), Always (3)

b Positive filler items were not considered for inclusion in the brief screening measure

c Item was reverse scored (Always=0, Often=1, Sometimes=2, Never=3)

d Interference item response options: No, not at all (0), Yes, only a little (1), Yes, quite a lot (2), Yes, a great deal (3)

e Response options: Less than a month (0), 1-5 months (1), 6-12 months (2), Over a year (3)

f Participant feedback that item was hard to understand or answer, and/or it was not considered relevant

Table S2 Summary of responses for parent-report symptom and impact items (73 items)

| **Item label** | **Item** |  | **N** |  | **Response percentage** | | | |
| --- | --- | --- | --- | --- | --- | --- | --- | --- |
|  |  |  |  |  | **0** | **1** | **2** | **3** |
|  | **Symptom items^a^** |  |  |  |  |  |  |  |
| worry_p | 1. My child worries about things |  | 645 |  | 3.9 | 54.7 | 31.5 | 9.9 |
| stom_p | 2. When my child has a problem, they get a funny feeling in their stomach |  | 634 |  | 33.1 | 39.9 | 17.8 | 9.1 |
| afraid_p | 3. My child complains of feeling afraid |  | 642 |  | 39.1 | 45.0 | 13.1 | 2.8 |
| alone_p | 4. My child would feel afraid of being on their own at home^e^ |  | 625 |  | 33.4 | 28.8 | 12.2 | 25.6 |
| test_p | 5. My child feels scared when they have to take a test |  | 646 |  | 27.7 | 45.0 | 14.7 | 12.5 |
| nerves_p | 6. My child feels nervous |  | 644 |  | 12.6 | 58.5 | 24.1 | 4.8 |
| toilet_p | 7. My child feels afraid if they have to use public toilets or bathrooms |  | 646 |  | 67.3 | 21.5 | 7.1 | 4.0 |
| awayp_p | 8. My child worries about being away from me/us |  | 644 |  | 31.1 | 45.3 | 13.0 | 10.6 |
| fool_p | 9. My child feels afraid that they will make a fool of themself in front of people |  | 645 |  | 27.1 | 43.1 | 18.1 | 11.6 |
| work_p | 10. My child worries that they will do badly at their school work |  | 644 |  | 26.7 | 42.7 | 17.5 | 13.0 |
| talkch_p | 11. My child talks in front of other children in their class**^b,^** ^e^ |  | 640 |  | 27.2 | 28.9 | 37.7 | 6.2 |
| awffam_p | 12. My child worries that something awful will happen to someone in our family |  | 644 |  | 37.6 | 41.9 | 14.0 | 6.5 |
| breath_p | 13. My child complains of suddenly feeling as if they can't breathe when there is no reason for this |  | 645 |  | 87.0 | 9.9 | 2.5 | 0.6 |
| check_p | 14. My child has to keep checking that they have done things right (like the switch is off, or the door is locked) |  | 645 |  | 75.7 | 15.0 | 5.6 | 3.7 |
| sleep_p | 15. My child is scared if they have to sleep on their own |  | 645 |  | 59.1 | 27.8 | 5.6 | 7.6 |
| school_p | 16. My child has trouble going to school in the mornings because they feel nervous or afraid |  | 646 |  | 71.5 | 21.1 | 5.3 | 2.2 |
| silly_p | 17. My child can't seem to get bad or silly thoughts out of their head |  | 642 |  | 44.1 | 38.3 | 13.6 | 4.0 |
| heart_p | 18. When my child has a problem, they complain of their heart beating really fast |  | 645 |  | 73.3 | 20.5 | 4.8 | 1.4 |
| trembl_p | 19. My child suddenly starts to tremble or shake when there is no reason for this |  | 645 |  | 90.7 | 8.1 | 0.8 | 0.5 |
| bad_p | 20. My child worries that something bad will happen to them |  | 645 |  | 45.7 | 44.3 | 7.1 | 2.8 |
| shaky_p | 21. When my child has a problem, they feel shaky |  | 642 |  | 74.0 | 21.8 | 2.8 | 1.4 |
| specl_p | 22. My child has to think of special thoughts (like numbers or words) to stop bad things from happening |  | 644 |  | 88.7 | 7.8 | 2.6 | 0.9 |
| stupid_p | 23. My child worries they might say or do something stupid in front of other children |  | 644 |  | 39.0 | 44.9 | 10.1 | 6.1 |
| travl_p | 24. My child feels scared if they have to travel in the car, or on a bus or a train |  | 644 |  | 83.4 | 12.6 | 3.1 | 0.9 |
| othink_p | 25. My child worries what other people think of them |  | 642 |  | 25.1 | 47.4 | 17.0 | 10.6 |
| crowd_p | 26. My child is afraid of being in crowded places (like shopping centres, the movies, buses, busy playgrounds) |  | 645 |  | 72.4 | 17.8 | 7.0 | 2.8 |
| scared_p | 27. All of a sudden my child feels really scared for no reason at all |  | 645 |  | 78.9 | 17.4 | 2.9 | 0.8 |
| dizzy_p | 28. My child complains of suddenly becoming dizzy or faint when there is no reason for this |  | 643 |  | 89.1 | 8.6 | 2.0 | 0.3 |
| front_p | 29. My child feels afraid if they have to talk in front of their class |  | 640 |  | 47.8 | 35.8 | 10.0 | 6.4 |
| wakeup_p | 30. My child wakes up feeling scared |  | 644 |  | 72.2 | 22.0 | 5.4 | 0.3 |
| hrt_nr_p | 31. My child complains of their heart suddenly starting to beat too quickly for no reason |  | 644 |  | 87.3 | 10.1 | 2.0 | 0.6 |
| wscare_p | 32. My child worries that they will suddenly get a scared feeling when there is nothing to be afraid of |  | 644 |  | 77.6 | 17.9 | 3.7 | 0.8 |
| small_p | 33. My child is afraid of being in small closed places, like tunnels or small rooms |  | 644 |  | 73.8 | 19.3 | 4.7 | 2.3 |
| repeat_p | 34. My child has to do some things over and over again (like washing their hands, cleaning or putting things in a certain order) |  | 645 |  | 84.5 | 9.6 | 4.2 | 1.7 |
| bother_p | 35. My child gets bothered by bad or silly thoughts or pictures in their mind |  | 642 |  | 53.6 | 33.0 | 10.0 | 3.4 |
| stpbad_p | 36. My child has to do certain things in just the right way to stop bad things happening |  | 645 |  | 88.8 | 6.8 | 3.4 | 0.9 |
| awayh_p | 37. My child would feel scared if they had to stay away from home overnight |  | 641 |  | 55.1 | 32.0 | 5.0 | 8.0 |
| poorly_p | 38. My child worries when they think they have done poorly at something |  | 644 |  | 23.4 | 49.2 | 17.1 | 10.2 |
| angry_p | 39. My child worries when they think someone is angry with them |  | 643 |  | 15.4 | 51.0 | 18.4 | 15.2 |
| flish_p | 40. My child worries about looking foolish |  | 644 |  | 32.0 | 46.1 | 13.0 | 8.9 |
| talkt_p | 41. My child talks in front of teachers**^b,^** ^e^ |  | 639 |  | 38.5 | 28.6 | 27.1 | 5.8 |
| awfp_p | 42. My child worries that bad things will happen to me |  | 642 |  | 39.9 | 41.1 | 11.1 | 7.9 |
| mistak_p | 43. My child worries about making mistakes |  | 645 |  | 20.2 | 53.8 | 15.5 | 10.5 |
| happen_p | 44. My child worries about what is going to happen |  | 643 |  | 30.9 | 44.2 | 16.2 | 8.7 |
| death_p | 45. My child thinks about death |  | 641 |  | 41.3 | 46.8 | 8.4 | 3.4 |
| wbed_p | 46. My child worries when they go to bed at night |  | 644 |  | 49.2 | 32.6 | 10.4 | 7.8 |
| qsttch_p | 47. When my child needs to, they ask their teacher questions**^b^** |  | 641 |  | 33.7 | 31.2 | 30.0 | 5.1 |
| stpwry_p | 48. My child finds it hard to stop worrying |  | 644 |  | 42.2 | 35.2 | 13.8 | 8.7 |
| newppl_p | 49. My child feels scared when they meet new people |  | 645 |  | 43.3 | 41.4 | 10.5 | 4.8 |
| embras_p | 50. My child is afraid they'll do something embarrassing |  | 642 |  | 33.5 | 47.8 | 12.1 | 6.5 |
| w_age_p | 51. My child worries about things more than other children their age^e^ |  | 637 |  | 49.9 | 26.1 | 14.8 | 9.3 |
| w_sit_p | 52. My child worries about things more than other children in a similar situation^e^ |  | 635 |  | 48.0 | 28.3 | 15.4 | 8.2 |
| avoid_p | 53. My child tries to avoid something because it scares them |  | 643 |  | 32.3 | 43.1 | 15.4 | 9.2 |
| else_p | 54. My child is afraid of something else (e.g. dogs, the dark, going to the doctor or dentist, being sick) |  | 643 |  | 36.7 | 36.7 | 15.2 | 11.4 |
|  | **Impact items^c^** |  |  |  |  |  |  |  |
| prob_p | 55. Do fears, worries or anxiety cause problems for your child? |  | 641 |  | 35.3 | 35.1 | 21.1 | 8.6 |
| howlong_p | 56. If yes, how long has your child had problems with fears, worries or anxiety?^d^ |  | 636 |  | 38.5 | 2.4 | 5.3 | 53.8 |
| upset_p | 57. Do fears, worries or anxiety upset or distress your child? |  | 641 |  | 26.5 | 41.7 | 20.0 | 11.9 |
| stop_p | 58. Do fears, worries or anxiety stop your child from doing things? |  | 640 |  | 42.2 | 36.2 | 15.0 | 6.6 |
| cfrds_p | 59. Do fears, worries or anxiety make these things difficult for your child's everyday life in the following areas? a. making friends or keeping friends |  | 642 |  | 58.3 | 22.6 | 13.4 | 5.8 |
| cfun_p | 60. Do fears, worries or anxiety make these things difficult for your child's everyday life in the following areas? b. having fun |  | 639 |  | 61.7 | 26.3 | 9.1 | 3.0 |
| cplaces_p | 61. Do fears, worries or anxiety make these things difficult for your child's everyday life in the following areas? c. going places |  | 639 |  | 61.5 | 26.0 | 7.0 | 5.5 |
| cwork_p | 62. Do fears, worries or anxiety make these things difficult for your child's everyday life in the following areas? d. doing schoolwork or homework |  | 639 |  | 57.1 | 23.9 | 11.1 | 7.8 |
| calongf_p | 63. Do fears, worries or anxiety make these things difficult for your child's everyday life in the following areas? e. getting along with family |  | 640 |  | 71.7 | 18.3 | 6.2 | 3.8 |
| csleep_p | 64. Do fears, worries or anxiety make these things difficult for your child's everyday life in the following areas? f. sleeping |  | 640 |  | 54.4 | 24.8 | 10.9 | 9.8 |
| clife_p | 65. Do fears, worries or anxiety make these things difficult for your child's everyday life in the following areas? g. life in general |  | 638 |  | 50.9 | 31.2 | 13.6 | 4.2 |
| fam_p | 66. Do your child's fears, worries or anxiety make things difficult for your family as a whole? |  | 641 |  | 53.2 | 27.9 | 13.9 | 5.0 |
| prelat_p | 67. Do your child's fears, worries or anxiety make these things difficult for your everyday life in the following areas? a. your relationships with family or friends |  | 643 |  | 72.8 | 18.7 | 5.9 | 2.6 |
| pwork_p | 68. Do your child's fears, worries or anxiety make these things difficult for your everyday life in the following areas? b. your career or work |  | 639 |  | 80.4 | 12.7 | 4.7 | 2.2 |
| pplaces_p | 69. Do your child's fears, worries or anxiety make these things difficult for your everyday life in the following areas? c. going places with or without your child |  | 642 |  | 66.2 | 19.2 | 9.7 | 5.0 |
| psleep_p | 70. Do your child's fears, worries or anxiety make these things difficult for your everyday life in the following areas? d. your sleep |  | 642 |  | 63.4 | 21.8 | 8.6 | 6.2 |
| pstress_p | 71. Do your child's fears, worries or anxiety make these things difficult for your everyday life in the following areas? e. your overall level of stress |  | 642 |  | 50.0 | 29.3 | 13.4 | 7.3 |
| cbenef_p | 72. Do you think your child would benefit from some support with fears, worries or anxiety? |  | 637 |  | 34.9 | 29.0 | 17.6 | 18.5 |
| pbenef_p | 73. Do you think you would benefit from some support to help your child overcome difficulties with fears, worries or anxiety? |  | 637 |  | 41.8 | 28.1 | 16.8 | 13.3 |

a Symptom item response options: Never (0), Sometimes (1), Often (2), Always (3)

b Item was reverse scored (Always=0, Often=1, Sometimes=2, Never=3)

c Interference item response options: No, not at all (0), Yes, only a little (1), Yes, quite a lot (2), Yes, a great deal (3)

d Response options: Less than a month (0), 1-5 months (1), 6-12 months (2), Over a year (3)

e Participant feedback that item was hard to understand or answer, and/or it was not considered relevant

Table S3 Summary of responses for teacher-report symptom and impact items (62 items)

|  | **Item** |  | **N** |  | **Response percentage** | | | | |
| --- | --- | --- | --- | --- | --- | --- | --- | --- | --- |
|  |  |  |  |  | **0** | **1** | **2** | **3** |  |
|  | **Symptom items^a^** |  |  |  |  |  |  |  |  |
| worry_t | 1. Worries about things |  | 561 |  | 16.6 | 58.6 | 19.8 | 5.0 |  |
| stom_t | 2. When they have a problem, they get a funny feeling in their stomach |  | 540 |  | 53.0 | 36.9 | 8.3 | 1.9 |  |
| afraid_t | 3. Complains of feeling afraid |  | 561 |  | 75.6 | 20.9 | 2.7 | 0.9 |  |
| test_t | 4. Feels scared when they have to take a test |  | 551 |  | 43.9 | 41.4 | 12.2 | 2.5 |  |
| nerves_t | 5. Feels nervous |  | 552 |  | 25.2 | 57.2 | 14.5 | 3.1 |  |
| toilet_t | 6. Feels afraid if they have to use school or public toilets or bathrooms |  | 551 |  | 96.0 | 3.6 | 0.4 | 0.0 |  |
| awayp_t | 7. Worries about being away from their parents |  | 546 |  | 71.1 | 24.5 | 3.1 | 1.3 |  |
| fool_t | 8. Feels afraid that they will make a fool of themself in front of people |  | 556 |  | 44.8 | 45.1 | 8.8 | 1.3 |  |
| work_t | 9. Worries that they will do badly at their school work |  | 552 |  | 30.8 | 50.4 | 15.6 | 3.3 |  |
| talkch_t | 10. Talks in front of other children in the class, when appropriate to do so^b^ |  | 565 |  | 41.8 | 33.3 | 21.8 | 3.2 |  |
| awffam_t | 11. Worries that something awful will happen to someone in their family |  | 527 |  | 82.7 | 12.1 | 4.4 | 0.8 |  |
| breath_t | 12. Complains of suddenly feeling as if they can't breathe when there is no reason for this |  | 558 |  | 95.9 | 3.9 | 0.2 | 0.0 |  |
| check_t | 13. Has to keep checking that they have done things right (like the switch is off, or the door is locked) |  | 555 |  | 84.9 | 12.3 | 2.3 | 0.5 |  |
| school_t | 14. Has trouble going to school in the mornings because they feel nervous or afraid |  | 541 |  | 83.9 | 12.6 | 2.8 | 0.7 |  |
| silly_t | 15. Can't seem to get bad or silly thoughts out of their head |  | 536 |  | 79.5 | 15.9 | 3.9 | 0.7 |  |
| heart_t | 16. When they have a problem, they complain of their heart beating really fast |  | 556 |  | 93.3 | 5.6 | 0.9 | 0.2 |  |
| trembl_t | 17. Suddenly starts to tremble or shake when there is no reason for this |  | 559 |  | 94.6 | 4.5 | 0.5 | 0.4 |  |
| bad_t | 18. Worries that something bad will happen to them |  | 538 |  | 77.9 | 19.7 | 2.2 | 0.2 |  |
| shaky_t | 19. When they have a problem, they feel shaky |  | 555 |  | 84.9 | 12.4 | 2.3 | 0.4 |  |
| specl_t | 20. Has to think of special thoughts to stop bad things from happening (like numbers or words) |  | 534 |  | 96.4 | 3.4 | 0.2 | 0.0 |  |
| stupid_t | 21. Worries they might say or do something stupid in front of other children |  | 552 |  | 47.1 | 44.0 | 7.4 | 1.4 |  |
| travl_t | 22. Feels scared if they have to travel in a car, or on a bus, minibus, coach or a train |  | 529 |  | 94.7 | 4.9 | 0.4 | 0.0 |  |
| othink_t | 23. Worries what other people think of them |  | 548 |  | 32.7 | 54.6 | 10.2 | 2.6 |  |
| crowd_t | 24. Is afraid of being in crowded places (like buses, busy playgrounds, the canteen) |  | 547 |  | 92.0 | 6.9 | 0.7 | 0.4 |  |
| scared_t | 25. All of a sudden feels really scared for no reason at all |  | 553 |  | 91.1 | 6.9 | 1.6 | 0.4 |  |
| dizzy_t | 26. Suddenly becomes dizzy or faint when there is no reason for this |  | 555 |  | 95.9 | 3.8 | 0.4 | 0.0 |  |
| front_t | 27. Feels afraid if they have to talk in front of the class |  | 562 |  | 58.0 | 33.8 | 6.2 | 2.0 |  |
| hrt_nr_t | 28. Complains of their heart suddenly starting to beat too quickly for no reason |  | 556 |  | 96.8 | 2.5 | 0.7 | 0.0 |  |
| wscare_t | 29. Worries that they will suddenly get a scared feeling when there is nothing to be afraid of |  | 551 |  | 91.1 | 7.4 | 1.3 | 0.2 |  |
| small_t | 30. Afraid of being in small closed places, like tunnels or small rooms. |  | 528 |  | 97.9 | 1.7 | 0.2 | 0.2 |  |
| repeat_t | 31. Has to do some things over and over again (like washing their hands, cleaning or putting things in a certain order) |  | 551 |  | 96.4 | 3.1 | 0.5 | 0.0 |  |
| bother_t | 32. Gets bothered by bad or silly thoughts or pictures in their mind |  | 539 |  | 83.9 | 13.4 | 2.2 | 0.6 |  |
| stpbad_t | 33. Has to do some things in just the right way to stop bad things happening |  | 544 |  | 96.3 | 3.3 | 0.4 | 0.0 |  |
| awayh_t | 34. Would feel scared if they had to stay away from home overnight |  | 496 |  | 74.4 | 21.6 | 3.0 | 1.0 |  |
| poorly_t | 35. Worries when they think they have done poorly at something |  | 552 |  | 33.3 | 51.1 | 13.2 | 2.4 |  |
| angry_t | 36. Feels worried when they think someone is angry with them |  | 547 |  | 34.2 | 47.9 | 14.6 | 3.3 |  |
| flish_t | 37. Worries they might look foolish |  | 550 |  | 40.2 | 49.5 | 8.2 | 2.2 |  |
| talkt_t | 38. Talks in front of teachers, when appropriate to do so**^b^** |  | 564 |  | 50.7 | 28.2 | 19.5 | 1.6 |  |
| awfslf_t | 39. Worries that bad things will happen to them |  | 538 |  | 74.3 | 22.7 | 2.2 | 0.7 |  |
| mistak_t | 40. Worries about making mistakes |  | 551 |  | 29.9 | 51.9 | 14.2 | 4.0 |  |
| happen_t | 41. Worries about what is going to happen |  | 543 |  | 52.7 | 39.0 | 6.1 | 2.2 |  |
| death_t | 42. Thinks about death |  | 523 |  | 84.5 | 13.6 | 1.3 | 0.6 |  |
| qsttch_t | 43. When they need to, they ask a teacher questions**^b^** |  | 563 |  | 42.3 | 28.1 | 24.3 | 5.3 |  |
| stpwry_t | 44. Finds it hard to stop worrying |  | 541 |  | 56.9 | 30.7 | 9.4 | 3.0 |  |
| newppl_t | 45. Feels scared when they meet new people |  | 537 |  | 75.0 | 22.9 | 1.3 | 0.7 |  |
| embras_t | 46. Afraid they'll do something embarrassing |  | 541 |  | 53.8 | 40.9 | 4.3 | 1.1 |  |
| w_age_t | 47. Worries about things more than other children their age |  | 552 |  | 58.0 | 25.7 | 11.6 | 4.7 |  |
| w_sit_t | 48. Worries about things more than other children in a similar situation |  | 551 |  | 59.7 | 25.4 | 11.3 | 3.6 |  |
| avoid_t | 49. Tries to avoid something because it scares them |  | 556 |  | 68.3 | 23.7 | 6.1 | 1.8 |  |
| else_t | 50. Afraid of something else (e.g. dogs, the dark, going to the doctor or dentist, being sick) |  | 526 |  | 82.5 | 14.4 | 1.5 | 1.5 |  |
|  | **Impact items^c^** |  |  |  |  |  |  |  |  |
| prob_t | 51. Do fears, worries or anxiety cause problems for this child? |  | 561 |  | 54.4 | 29.1 | 12.7 | 3.9 |  |
| howlong_t | 52. If yes, how long has this child had problems with fears, worries or anxiety?^d^ |  | 545 |  | 58.2 | 10.5 | 5.0 | 26.4 |  |
| upset_t | 53. Do fears, worries or anxiety upset or distress this child? |  | 559 |  | 51.9 | 30.8 | 13.2 | 4.1 |  |
| stop_t | 54. Do fears, worries or anxiety stop this child from doing things? |  | 559 |  | 67.4 | 21.6 | 7.9 | 3.0 |  |
| cfrds_t | 55. Do fears, worries or anxiety make things difficult for this child's everyday life in the following areas? 4a. making friends or keeping friends |  | 558 |  | 71.9 | 18.5 | 7.9 | 1.8 |  |
| cfun_t | 56. Do fears, worries or anxiety make things difficult for this child's everyday life in the following areas?4b. having fun |  | 558 |  | 75.8 | 18.5 | 4.8 | 0.9 |  |
| cplaces_t | 57. Do fears, worries or anxiety make things difficult for this child's everyday life in the following areas?4c. going places |  | 542 |  | 87.1 | 8.5 | 3.5 | 0.9 |  |
| cwork_t | 58. Do fears, worries or anxiety make things difficult for this child's everyday life in the following areas? 4d. doing schoolwork or homework |  | 558 |  | 67.6 | 22.6 | 7.2 | 2.7 |  |
| calongf_t | 59. Do fears, worries or anxiety make things difficult for this child's everyday life in the following areas? 4e. getting along with family |  | 523 |  | 87.2 | 9.2 | 2.9 | 0.8 |  |
| clife_t | 60. Do fears, worries or anxiety make things difficult for this child's everyday life in the following areas?4f. life in general |  | 538 |  | 71.0 | 21.2 | 6.7 | 1.1 |  |
| class_t | 61. Do this child's fears, worries or anxiety make things difficult for you or the class as a whole? |  | 559 |  | 78.9 | 15.0 | 4.7 | 1.4 |  |
| cbenef_t | 62. Do you think this child would benefit from some support with fears, worries or anxiety? |  | 556 |  | 46.0 | 32.6 | 13.1 | 8.3 |  |

a Symptom item response options: Never (0), Sometimes (1), Often (2), Always (3)

b Item was reverse scored (Always=0, Often=1, Sometimes=2, Never=3)

c Interference item response options: No, not at all (0), Yes, only a little (1), Yes, quite a lot (2), Yes, a great deal (3)

d Response options: Less than a month (0), 1-5 months (1), 6-12 months (2), Over a year (3)

Table S4 Exploratory factor analysis of 66 child-report items (symptoms and impact):Oblique rotated factor loadings for 6 salient factors

| Item | Factor 1 | Factor 2 | Factor 3 | Factor 4 | Factor 5 | Factor 6 |
| --- | --- | --- | --- | --- | --- | --- |
| worry_c | 0.433*^a^ | 0.261* | 0.193* | 0.105 | -0.105* | -0.165* |
| stom_c | 0.2 | 0.441* ^a^ | 0.047 | 0.114* | -0.091* | -0.291* |
| afraid_c | 0.434* ^a^ | 0.268* | 0.108 | 0.014 | 0.07 | -0.380* |
| alone_c | 0.440* ^a^ | -0.098 | -0.005 | 0.519* ^a^ | -0.036 | -0.039 |
| test_c | -0.104 | 0.187* | 0.521* ^a^ | 0.212* | -0.004 | -0.075 |
| nerves_c | 0.232 | 0.371* | 0.189* | 0.054 | -0.018 | -0.312* |
| toilet_c | 0.025 | 0.344* | 0.196* | 0.235* | -0.027 | -0.003 |
| **awayp_c** | 0.473* ^a^ | 0.05 | -0.048 | **0.563*^b^** | -0.002 | -0.083 |
| fool_c | 0.066 | -0.031 | 0.747* ^a^ | -0.027 | 0.037 | -0.120* |
| work_c | 0.017 | 0.043 | 0.676* ^a^ | 0.046 | -0.018 | 0.042 |
| talkch_c | -0.028 | -0.016 | 0.083 | 0.169* | 0.620* ^a^ | 0.163 |
| awffam_c | 0.653* ^a^ | 0.095 | -0.001 | 0.062 | 0.123* | 0.002 |
| breath_c | 0.213* | 0.607* ^a^ | -0.019 | 0.011 | 0.134* | -0.025 |
| check_c | -0.043 | 0.495* ^a^ | 0.075 | 0.069 | 0.005 | 0.221* |
| sleep_c | 0.704* ^a^ | -0.013 | -0.193* | 0.405* ^a^ | 0.146* | -0.022 |
| school_c | 0.283* | 0.350* | 0.169* | 0.169* | 0.057 | -0.071 |
| silly_c | 0.470* ^a^ | 0.276* | 0.009 | -0.073 | -0.094* | 0.068 |
| heart_c | 0.033 | 0.597* ^a^ | 0.08 | -0.05 | -0.084 | -0.003 |
| **trembl_c** | 0.067 | **0.793*^b^** | -0.093 | -0.048 | 0.027 | -0.006 |
| bad_c | 0.861* ^a^ | -0.026 | 0.09 | -0.073 | 0.228* | -0.017 |
| shaky_c | -0.019 | 0.609* ^a^ | 0.203* | 0.034 | -0.007 | -0.057 |
| specl_c | 0.193* | 0.433* ^a^ | 0.02 | 0.073 | 0.043 | 0.130* |
| stupid_c | 0.194 | 0.05 | 0.709* ^a^ | -0.135* | 0.033 | -0.073 |
| travl_c | 0.192* | 0.345* | 0.105 | 0.236* | 0.029 | 0.049 |
| othink_c | 0.237 | 0.009 | 0.700* ^a^ | -0.183* | 0.011 | -0.036 |
| crowd_c | 0.036 | 0.432* ^a^ | 0.126 | 0.284* | 0.026 | 0.112* |
| scared_c | 0.394* | 0.569* ^a^ | 0.007 | -0.021 | 0.034 | -0.052 |
| dizzy_c | 0.065 | 0.701* ^a^ | -0.01 | -0.119 | 0.019 | 0.105 |
| front_c | -0.025 | 0.075 | 0.562* ^a^ | 0.205* | 0.279* | 0.062 |
| wakeup_c | 0.525* ^a^ | 0.248* | 0.002 | 0.087 | 0.02 | -0.069 |
| hrt_nr_c | 0.231* | 0.749* ^a^ | -0.058 | -0.228* | -0.039 | 0.095 |
| wscare_c | 0.548* ^a^ | 0.392* | -0.024 | 0.018 | 0.075 | -0.073 |
| small_c | -0.051 | 0.374* | 0.179* | 0.296* | -0.012 | 0.028 |
| repeat_c | -0.07 | 0.485* ^a^ | 0.08 | 0.048 | 0.022 | 0.264* |
| bother_c | 0.574* ^a^ | 0.141* | 0.059 | -0.058 | -0.069 | 0.140* |
| stpbad_c | 0.14 | 0.492* ^a^ | -0.074 | -0.01 | 0.100* | 0.193* |
| awayh_c | 0.332* | 0.056 | 0.036 | 0.470* ^a^ | 0.003 | 0.03 |
| poorly_c | 0.142 | -0.042 | 0.618* ^a^ | 0.028 | -0.202* | 0.160* |
| angry_c | 0.211* | 0.053 | 0.477* ^a^ | 0.145* | -0.154* | 0.018 |
| **flish_c** | 0.209 | -0.174* | **0.826*^b^** | -0.167* | 0.039 | -0.034 |
| **talkt_c** | 0.001 | 0.073 | 0.02 | 0.043 | **0.706* ^b^** | 0.114 |
| **awfslf_c** | **0.878*^b^** | -0.005 | 0.071 | -0.055 | 0.220* | 0.022 |
| mistak_c | -0.058 | 0.149* | 0.711* ^a^ | 0.021 | -0.143* | 0.081 |
| happen_c | 0.540* ^a^ | 0.058 | 0.301* | -0.101* | -0.054 | 0.087 |
| death_c | 0.688* ^a^ | 0.029 | 0.011 | -0.190* | 0.012 | 0.05 |
| wbed_c | 0.803* ^a^ | -0.091 | -0.048 | 0.321* | 0.038 | -0.033 |
| qsttch_c | 0.001 | 0.216* | -0.008 | -0.038 | 0.470* ^a^ | -0.074 |
| stpwry_c | 0.505* ^a^ | 0.306* | 0.139* | 0 | -0.135* | -0.057 |
| newppl_c | 0.08 | 0.073 | 0.407* ^a^ | 0.292* | -0.008 | 0.144* |
| embras_c | 0.177 | -0.142* | 0.800* ^a^ | 0 | 0.044 | 0.045 |
| w_age_c | 0.415* ^a^ | 0.297* | 0.200* | -0.057 | -0.101* | -0.002 |
| avoid_c | 0.453* ^a^ | 0.05 | 0.159* | 0.115 | -0.079 | 0.02 |
| else_c | 0.347* | 0.031 | 0.203* | 0.271* | -0.143* | 0.034 |
| prob_c | 0.587* ^a^ | 0.212* | 0.048 | 0.031 | -0.130* | 0.067 |
| howlong_c | 0.631* ^a^ | 0.063 | 0.082 | 0.002 | -0.182* | -0.009 |
| upset_c | 0.555* ^a^ | 0.175* | 0.083 | 0.048 | -0.168* | 0.107* |
| stop_c | 0.415* ^a^ | 0.222* | 0.119* | -0.135* | 0.007 | 0.158* |
| cfrds_c | 0.19 | 0.117 | 0.324* | 0.023 | 0.028 | 0.281* |
| cfun_c | 0.324* | 0.092 | 0.104 | -0.023 | 0.008 | 0.453* ^a^ |
| cplaces_c | 0.290* | 0.131 | 0.093 | 0.144* | 0.098* | 0.348* |
| cwork_c | 0.021 | 0.259* | 0.332* | 0.134* | 0.049 | 0.331* |
| **calongf_c** | 0.332* | 0.023 | 0.026 | -0.051 | 0.005 | **0.572*^b^** |
| csleep_c | 0.747* ^a^ | -0.05 | -0.097 | 0.121* | -0.035 | 0.279* |
| clife_c | 0.586* ^a^ | -0.033 | 0.044 | -0.016 | -0.042 | 0.415* ^a^ |
| fam_c | 0.409* ^a^ | 0.299* | -0.052 | -0.048 | -0.045 | 0.258* |
| chelp_c | 0.594* ^a^ | 0.083 | 0.026 | 0.142* | -0.168* | 0.041 |

*p<0.05

a factor loading >0.4

b highest loading for the factor

Table S5 Exploratory factor analysis of 73 parent-report items (symptoms and impact items): Oblique rotated factor loadings for 7 salient factors

| Item | Factor 1 | Factor 2 | Factor 3 | Factor 4 | Factor 5 | Factor 6 | Factor 7 |
| --- | --- | --- | --- | --- | --- | --- | --- |
| worry_p | 0.061 | 0.531* ^a^ | 0.200* | 0.137 | -0.136* | 0.035 | -0.255* |
| stom_p | 0.052 | 0.343* | 0.291* | 0.014 | -0.056 | 0.106* | -0.206* |
| afraid_p | 0.088 | 0.604* ^a^ | 0.147* | -0.031 | 0.140* | 0.097 | -0.096* |
| alone_p | 0.057 | 0.365* | 0.141* | -0.039 | 0.011 | 0.265* | -0.074 |
| test_p | 0.291* | -0.071 | 0.493* ^a^ | 0.132 | 0.002 | 0.021 | -0.299* |
| nerves_p | 0.228* | 0.319* | 0.249* | 0.129 | -0.083 | 0.062 | -0.300* |
| toilet_p | 0.136* | 0.122 | 0.073 | 0.162 | 0.078 | 0.328* | -0.026 |
| awayp_p | -0.063 | 0.327* | 0.255* | 0.007 | 0.018 | 0.399* | -0.03 |
| fool_p | 0.838*^a^ | 0.101 | -0.022 | -0.042 | 0.066 | 0.015 | 0.061 |
| work_p | 0.482* ^a^ | 0.01 | 0.496* ^a^ | 0.056 | -0.023 | -0.133* | -0.290* |
| talkch_p | 0.446* ^a^ | -0.096 | 0.004 | 0.039 | 0.713* ^a^ | 0.127* | -0.053 |
| awffam_p | 0.005 | 0.364* | 0.477* ^a^ | -0.078 | -0.128* | -0.054 | 0.279* |
| breath_p | -0.041 | 0.223* | 0.558* ^a^ | -0.005 | 0.085 | 0.103 | 0.124 |
| check_p | -0.012 | -0.043 | 0.150* | 0.323* | -0.086 | 0.167 | 0.510* ^a^ |
| sleep_p | -0.034 | 0.641* ^a^ | 0.001 | -0.005 | 0.280* | 0.109 | 0.038 |
| school_p | 0.081 | 0.262* | 0.180* | 0.350* | 0.051 | 0.105* | -0.121* |
| silly_p | 0.105 | 0.646* ^a^ | 0.016 | 0.102 | -0.006 | -0.092 | 0.329* |
| heart_p | 0.053 | 0.048 | 0.730* ^a^ | 0.015 | 0.263* | 0.071 | 0.012 |
| trembl_p | -0.024 | 0.079 | 0.296* | 0.475* ^a^ | 0.234* | -0.042 | 0.188* |
| bad_p | 0.111* | 0.582* ^a^ | 0.186* | -0.035 | -0.079 | 0.032 | 0.278* |
| shaky_p | 0.086 | 0.127 | 0.441* ^a^ | 0.077 | 0.102 | 0.028 | 0.038 |
| specl_p | -0.006 | 0.155 | 0.142 | 0.255* | 0.034 | 0.12 | 0.479* ^a^ |
| **stupid_p** | **0.889*** ^a, b^ | 0.095 | 0.017 | 0.001 | 0.051 | -0.100* | 0.123* |
| travl_p | -0.038 | 0.083 | 0.12 | 0.192 | -0.072 | 0.451* ^a^ | 0.042 |
| othink_p | 0.715* ^a^ | 0.161* | 0.035 | 0.02 | -0.157* | -0.003 | 0.035 |
| crowd_p | 0.152* | 0.023 | 0.037 | 0.305* | 0.083 | 0.431* ^a^ | 0.163* |
| scared_p | -0.082 | 0.579* ^a^ | 0.191* | 0.135 | 0.061 | 0.144* | 0.153* |
| dizzy_p | -0.182* | 0.118 | 0.491* ^a^ | 0.241* | 0.067 | 0.13 | 0.098 |
| front_p | 0.639* ^a^ | 0.009 | 0.156* | -0.069 | 0.397* | 0.188* | -0.102* |
| wakeup_p | -0.036 | 0.662* ^a^ | 0.152* | -0.045 | 0.186* | 0.061 | 0.016 |
| **hrt_nr_p** | -0.023 | 0.064 | **0.826* ^a, b^** | 0.02 | 0.297* | 0.123 | 0.03 |
| wscare_p | 0.024 | 0.588* ^a^ | 0.108 | 0.088 | 0.131* | 0.194* | 0.109 |
| small_p | 0.148* | -0.036 | 0.106 | -0.009 | 0.08 | 0.463* ^a^ | 0.228* |
| repeat_p | -0.024 | -0.093 | -0.09 | 0.582* ^a^ | -0.043 | 0.155 | 0.591* ^a^ |
| bother_p | 0.093 | 0.698* ^a^ | 0.05 | 0.051 | -0.04 | -0.089 | 0.339* |
| **stpbad_p** | -0.01 | 0.026 | 0.026 | 0.467* ^a^ | -0.016 | 0.116 | **0.642* ^a, b^** |
| **awayh_p** | 0.073 | 0.189 | 0.181* | 0.041 | 0.039 | **0.487* ^a, b^** | -0.018 |
| poorly_p | 0.520* ^a^ | -0.021 | 0.423* ^a^ | 0.141* | -0.192* | -0.067 | -0.056 |
| angry_p | 0.351* | 0.171* | 0.305* | 0.072 | -0.139* | 0.061 | 0.018 |
| flish_p | 0.818* ^a^ | 0.129 | -0.001 | -0.031 | -0.051 | 0.049 | 0.119* |
| **talkt_p** | 0.461* ^a^ | -0.078 | -0.096 | 0.036 | **0.800*** ^a, b^ | -0.007 | -0.019 |
| awfp_p | 0.013 | 0.470* ^a^ | 0.388* | -0.046 | -0.104 | -0.025 | 0.339* |
| mistak_p | 0.577* ^a^ | -0.007 | 0.430* ^a^ | 0.045 | -0.148* | -0.106* | -0.025 |
| happen_p | 0.072 | 0.509* ^a^ | 0.228* | 0.131* | -0.225* | 0.024 | 0.038 |
| death_p | 0.111* | 0.402* ^a^ | 0.199* | -0.023 | -0.091 | -0.149* | 0.247* |
| **wbed_p** | -0.062 | **0.988* ^a, b^** | -0.037 | -0.048 | 0.191* | -0.068 | 0.018 |
| qsttch_p | 0.397* | 0.052 | 0 | 0.086 | 0.536* ^a^ | -0.046 | -0.028 |
| stpwry_p | 0.088* | 0.637* ^a^ | 0.06 | 0.172* | -0.122* | 0.082 | -0.006 |
| newppl_p | 0.461* ^a^ | 0.017 | 0.06 | 0.081 | 0.084* | 0.379* | 0.026 |
| embras_p | 0.882* ^a^ | 0.115 | -0.125* | -0.006 | 0.001 | 0.099* | 0.177* |
| w_age_p | 0.117* | 0.543* ^a^ | -0.097 | 0.340* | -0.203* | 0.248* | -0.034 |
| w_sit_p | 0.143* | 0.533* ^a^ | -0.161* | 0.338* | -0.213* | 0.264* | -0.056 |
| avoid_p | 0.218* | 0.310* | -0.077 | 0.216* | 0.027 | 0.299* | 0.031 |
| else_p | -0.022 | 0.396* | -0.016 | 0.114 | 0.033 | 0.296* | 0.034 |
| prob_p | -0.044 | 0.528* ^a^ | 0.036 | 0.505* ^a^ | -0.078* | 0.048 | -0.243* |
| howlong_p | 0.008 | 0.521* ^a^ | -0.004 | 0.459* ^a^ | -0.099* | 0.007 | -0.295* |
| upset_p | 0.003 | 0.496* ^a^ | 0.07 | 0.464* ^a^ | -0.035 | 0.021 | -0.138* |
| stop_p | 0.134* | 0.281* | -0.044 | 0.550* ^a^ | 0.061 | 0.108* | -0.021 |
| cfrds_p | 0.170* | 0.048 | 0.062 | 0.624* ^a^ | 0.012 | 0.079 | -0.012 |
| cfun_p | 0.186* | 0.056 | -0.001 | 0.649* ^a^ | -0.018 | 0.162* | -0.067 |
| cplaces_p | -0.023 | 0.068 | 0.008 | 0.610* ^a^ | -0.015 | 0.444* ^a^ | -0.062 |
| cwork_p | 0.236* | -0.085 | 0.306* | 0.503* ^a^ | 0.005 | -0.015 | -0.145* |
| calongf_p | 0.001 | 0.013 | 0.035 | 0.833* ^a^ | -0.018 | -0.098 | 0.101 |
| csleep_p | -0.123* | 0.867* ^a^ | -0.07 | 0.211* | 0.242* | -0.166* | 0.038 |
| clife_p | 0.06 | 0.243* | 0.06 | 0.652* ^a^ | -0.005 | 0.088* | 0.013 |
| fam_p | -0.045 | 0.265* | -0.004 | 0.772* ^a^ | 0.032 | 0.008 | -0.017 |
| **prelat_p** | 0.068 | -0.046 | 0.055 | **0.889* ^a, b^** | 0.024 | -0.035 | 0.170* |
| pwork_p | 0.096 | -0.018 | -0.031 | 0.843* ^a^ | 0.028 | -0.043 | 0.155* |
| pplaces_p | -0.043 | 0.057 | 0.031 | 0.735* ^a^ | -0.033 | 0.263* | 0.06 |
| psleep_p | -0.048 | 0.403* ^a^ | 0.077 | 0.534* ^a^ | 0.164* | -0.111* | 0.087 |
| pstress_p | 0.025 | 0.252* | 0.038 | 0.740* ^a^ | 0.048 | -0.112* | 0.025 |
| cbenef_p | 0.033 | 0.523* ^a^ | 0.014 | 0.583* ^a^ | 0.018 | -0.270* | -0.100* |
| pbenef_p | 0.016 | 0.507* ^a^ | -0.015 | 0.575* ^a^ | 0.066* | -0.311* | -0.072 |

*p<0.05

a factor loading >0.4

b highest loading for the factor

Table S6 Exploratory factor analysis of 62 teacher-report items (symptoms and impact items): Oblique rotated factor loadings for 6 salient factors

| Item | Factor 1 | Factor 2 | Factor 3 | Factor 4 | Factor 5 | Factor 6 |
| --- | --- | --- | --- | --- | --- | --- |
| worry_t | 0.266* | 0.163* | 0.206* | 0.409* ^a^ | -0.053 | 0.129 |
| stom_t | 0.491*^a^ | 0.290* | 0.095 | 0.065 | 0.011 | -0.046 |
| afraid_t | 0.513* ^a^ | 0.352* | 0.141* | 0.025 | -0.057 | -0.005 |
| test_t | 0.210* | 0.204* | 0.344* | 0.087 | 0.011 | 0.298* |
| nerves_t | 0.200* | 0.276* | 0.311* | 0.107 | 0.095 | 0.226* |
| toilet_t | 0.409* ^a^ | 0.205 | -0.031 | 0.025 | 0.291* | 0.168 |
| awayp_t | 0.528* ^a^ | 0.271 | 0.025 | 0.03 | 0.104 | 0.072 |
| fool_t | 0.042 | 0.127 | 0.691* ^a^ | 0.054 | 0.249* | 0.059 |
| work_t | 0 | 0.019 | 0.435* ^a^ | 0.113 | -0.022 | 0.578* ^a^ |
| talkch_t | 0.061 | -0.044 | 0.121 | 0.041 | 0.797* ^a^ | -0.072 |
| awffam_t | 0.818* ^a^ | 0.048 | -0.163* | 0.035 | 0.101 | -0.059 |
| breath_t | 0.355* | 0.710* ^a^ | -0.169 | -0.122 | -0.178 | 0.074 |
| check_t | 0.093 | 0.187 | 0.094 | -0.008 | 0.029 | 0.321* |
| school_t | 0.355* | 0.276* | -0.069 | 0.304* | 0.14 | 0.055 |
| silly_t | 0.899* ^a^ | -0.231* | -0.076 | 0.196* | -0.057 | 0.072 |
| heart_t | -0.018 | 0.949* ^a^ | 0.047 | 0.077 | -0.122 | -0.182 |
| trembl_t | 0.035 | 0.674* ^a^ | 0.162 | 0.033 | 0.053 | 0.033 |
| bad_t | 0.872* ^a^ | 0.048 | -0.006 | 0.024 | -0.004 | -0.047 |
| shaky_t | 0.146 | 0.678* ^a^ | 0.126 | 0.033 | -0.094 | 0.034 |
| specl_t | 0.680* ^a^ | 0.183 | -0.349* | 0.186 | 0.07 | -0.029 |
| **stupid_t** | 0.006 | -0.017 | **0.835* ^a, b^** | 0 | 0.255* | -0.02 |
| travl_t | 0.255 | 0.557* ^a^ | 0.158 | -0.217 | 0.045 | 0.215 |
| othink_t | -0.075 | -0.018 | 0.784* ^a^ | 0.275* | 0.038 | -0.068 |
| crowd_t | 0.319* | 0.469* ^a^ | 0.052 | 0.073 | 0.293* | 0.065 |
| scared_t | 0.455* ^a^ | 0.410* ^a^ | 0.117 | 0.123 | 0.014 | -0.089 |
| dizzy_t | 0.037 | 0.880* ^a^ | -0.285 | 0.15 | -0.181 | -0.223 |
| front_t | 0.068 | -0.005 | 0.535* ^a^ | 0.018 | 0.534* | -0.031 |
| hrt_nr_t | -0.075 | **1.138* ^a, b^** | -0.053 | 0.044 | -0.063 | -0.183 |
| wscare_t | 0.410* ^a^ | 0.394* | 0.170* | 0.026 | 0.161* | -0.034 |
| small_t | 0.379* | 0.614* ^a^ | 0.048 | -0.183 | 0.108 | 0.042 |
| repeat_t | -0.097 | 0.295 | -0.074 | 0.403* ^a^ | 0.209 | 0.345* |
| bother_t | 0.915* ^a^ | -0.216 | 0.013 | 0.057 | -0.035 | 0.008 |
| stpbad_t | 0.323* | 0.271 | -0.037 | -0.058 | 0.153 | 0.392* |
| awayh_t | 0.496* ^a^ | 0.281* | 0.052 | -0.198* | 0.093 | 0.134 |
| **poorly_t** | -0.026 | -0.03 | 0.37 | 0.062 | -0.21 | **0.774* ^a, b^** |
| angry_t | 0.103 | 0.026 | 0.414* ^a^ | 0.078 | -0.160* | 0.470* ^a^ |
| flish_t | -0.082 | 0.07 | 0.823* ^a^ | -0.024 | 0.223 | 0.065 |
| **talkt_t** | -0.013 | -0.061 | -0.002 | 0.012 | **0.911* ^a, b^** | 0.028 |
| **awfslf_t** | **0.949* ^a, b^** | -0.036 | 0.069 | -0.05 | -0.032 | -0.041 |
| mistak_t | 0.036 | 0.023 | 0.461* ^a^ | 0.077 | -0.175* | 0.544* ^a^ |
| happen_t | 0.537* ^a^ | 0.085 | 0.292* | 0.101 | -0.051 | 0.131 |
| death_t | 0.739* | 0.037 | -0.034 | -0.093 | 0.023 | -0.167* |
| qsttch_t | -0.078 | -0.024 | -0.033 | 0.116 | 0.834* ^a^ | -0.097 |
| stpwry_t | 0.447* ^a^ | 0.036 | 0.228* | 0.362* | -0.113* | 0.121 |
| newppl_t | 0.255 | 0.336* | 0.292* | -0.058 | 0.268* | 0.063 |
| embras_t | 0.007 | 0.195* | 0.794* ^a^ | -0.065 | 0.256* | 0.024 |
| w_age_t | 0.515* ^a^ | -0.054 | 0.324* | 0.499* ^a^ | -0.067 | -0.141 |
| w_sit_t | 0.460* ^a^ | -0.112* | 0.344* | 0.526* ^a^ | -0.089 | -0.065 |
| avoid_t | 0.241* | 0.213* | 0.276* | 0.264* | 0.248* | 0.078 |
| else_t | 0.360* | 0.376* | -0.01 | 0.045 | 0.045 | 0.136 |
| prob_t | 0.031 | 0.225* | 0.063 | 0.814* ^a^ | -0.053 | 0.025 |
| howlong_t | -0.048 | 0.283* | 0.185* | 0.717* ^a^ | -0.019 | -0.104 |
| upset_t | 0.106* | 0.124* | 0.022 | 0.768* ^a^ | -0.114 | 0.133* |
| stop_t | 0.065 | 0.071 | 0.09 | 0.696* ^a^ | 0.127 | 0.199* |
| cfrds_t | -0.04 | 0.005 | 0.036 | 0.781* ^a^ | 0.144 | -0.029 |
| cfun_t | 0.139 | 0.024 | 0.081 | 0.724* ^a^ | 0.145 | -0.014 |
| cplaces_t | 0.390* | 0.243* | -0.065 | 0.320* | 0.164 | 0.128 |
| cwork_t | 0.06 | -0.141 | 0.082 | 0.571* | 0.204 | 0.361* |
| calongf_t | 0.218* | 0.027 | -0.159* | 0.559* | 0.192 | 0.152 |
| clife_t | 0.211* | 0.025 | -0.085* | 0.764* ^a^ | 0.167 | 0.059 |
| **class_t** | -0.024 | -0.016 | -0.028 | **0.850* ^a, b^** | 0.044 | 0.206* |
| cbenef_t | 0.088 | 0.08 | 0.049 | 0.740* ^a^ | 0.102 | 0.071 |

*p<0.05

a factor loading >0.4

b highest loading for the factor

Table S7 Summary of factors identified in exploratory factor analysis (symptoms and impact items)

| Child-report | **Factor 1** – 'generalised anxiety’ (explains 42.5% of the variance)  Highest loading item (0.878): *I worry that bad things will happen to me*  **Factor 2** – ‘physical symptoms’ (explains 4.0% of the variance)  Highest loading item (0.793): *I suddenly start to tremble or shake when there is no reason for this*  **Factor 3** – ‘social anxiety’ (explains 3.7% of the variance)  Highest loading item (0.826): *I worry I might look foolish*  **Factor 4** – 'separation anxiety’ (explains 3.7% of the variance)  Highest loading item (0.563): *I worry about being away from my parents*  **Factor 5** – ‘talking in class’ (explains 2.7% of the variance)  Highest loading item (0.706): *I talk in front of my teachers*  **Factor 6** – 'impact’ (explains 2.5% of the variance)  Highest loading item (0.572): *Do fears or worries mess things up for you in these areas?... getting along with family* |
| --- | --- |
| Parent-report | **Factor 1** – ‘social anxiety’ (explains 47.8% of the variance).  Highest loading item (0.889): *My child worries they might say or do something stupid in front of other children*  **Factor 2** – ‘generalised anxiety’ (explains 5.6% of the variance).  Highest loading item (0.988): *My child worries when they go to bed at night*  **Factor 3** – ‘physical symptoms’ (explains 4.1% of the variance)  Highest loading item (0.826): *My child complains of their heart suddenly starting to beat too quickly for no reason*  **Factor 4** – ‘impact’ (explains 3.8% of the variance).  Highest loading item (0.889): *Do your child's fears, worries or anxiety make these things difficult for your everyday life….your relationships with family or friends*  **Factor 5** – ‘talking in class’ (explains 2.8% of the variance).  Highest loading item (0.800): *My child talks in front of teachers*  **Factor 6** – ‘outside home’ (explains 2.5% of the variance).  Highest loading item (0.487): *My child would feel scared if they had to stay away from home overnight*  **Factor 7** – ‘obsessive/compulsive behaviour’ (explains 2.4% of the variance).  Highest loading item (0.642): *My child has to do certain things in just the right way to stop bad things happening* |
| Teacher-report | **Factor 1** – ‘generalised anxiety’ (explains 51.8% of the variance).  Highest loading item (0.949): *Worries that bad things will happen to them*  **Factor 2** – ‘physical symptoms’ (explains 7.7% of the variance).  Highest loading item (1.138): *Complains of their heart suddenly starting to beat too quickly for no reason*  **Factor 3** – ‘social anxiety’ (explains 5.1% of the variance).  Highest loading item (0.835):  *Worries they might say or do something stupid in front of other children*  **Factor 4** – ‘interference’ (explains 4.9% of the variance).  Highest loading item (0.850): *Do this child's fears, worries or anxiety make things difficult for you or the class as a whole?*  **Factor 5** – ‘talking in class’ (explains 3.2% of the variance).  Highest loading item (0.911): *Talks in front of teachers, when appropriate to do so*  **Factor 6** – ‘school performance worries’ (explains 2.9% of the variance).  Highest loading item (0.774): *Worries when they think they have done poorly at something* |

Table S8 Indicators of fit for exploratory factor analysis models (symptom and impact items)

| Analysis | N | Number of items | Number of factors | Chi-squared (df) | RMSEA (90% CI) | CFI | TLI | SRMR |
| --- | --- | --- | --- | --- | --- | --- | --- | --- |
| Child-report | 511 | 66 | 6 | 2614.6 (1764) | 0.031 (0.028 to 0.033) | 0.977 | 0.972 | 0.037 |
| Parent-report | 548 | 73 | 7 | 3619.3 (2138) | 0.036 (0.034 to 0.038) | 0.978 | 0.973 | 0.041 |
| Teacher-report | 439 | 62 | 6 | 1966.3 (1534) | 0.025 (0.022 to 0.029) | 0.989 | 0.987 | 0.053 |

RMSEA – Root mean square error of approximation

CFI – Comparative Fit Index

TLI – Tucker-Lewis Index

SRMR – Standardised Root Mean Square Residual

Table S9 Identifying best predictors of anxiety disorder status: Backward elimination stepwise logistic regression (symptoms and impact)

| Reporter | Model | Items | Area Under the Curve |
| --- | --- | --- | --- |
| Child-report | 1 | *1. I worry that bad things will happen to me*  *2.I suddenly start to tremble or shake when there is no reason for this*  *3.I worry I might look foolish*  *4.I worry about being away from my parents*  *5.I talk in front of my teachers*  *6.Do fears or worries mess things up for you in these areas? 4e. getting along with family* |  |
|  | 2 | *1. I worry that bad things will happen to me*  *2.I suddenly start to tremble or shake when there is no reason for this* | 0.6956 |
|  | 3 | *1. I worry that bad things will happen to me*  *2. I suddenly start to tremble or shake when there is no reason for this*  *3. I have trouble going to school in the mornings because I feel nervous or afraid* ^a^  *4. I worry about things more than other children my age* ^a^  5. *I feel afraid if I have to talk in front of my class* ^a^ | 0.7938 |
| Parent-report | 1 | *1.My child worries they might say or do something stupid in front of other children*  *2.My child worries when they go to bed at night*  *3.My child complains of their heart suddenly starting to beat too quickly for no reason*  *4. Do your child's fears, worries or anxiety make these things difficult for your everyday life in the following areas? a. your relationships with family or friends*  *5.My child talks in front of teachers*  *6.My child would feel scared if they had to stay away from home overnight*  *7.My child has to do certain things in just the right way to stop bad things happening* |  |
|  | 2 | *1.My child worries they might say or do something stupid in front of other children*  *2.My child worries when they go to bed at night*  *3.My child complains of their heart suddenly starting to beat too quickly for no reason*  *4. Do your child's fears, worries or anxiety make these things difficult for your everyday life in the following areas? a. your relationships with family or friends*  *5.My child would feel scared if they had to stay away from home overnight* | 0.8280 |
|  | 3 | *1.My child worries they might say or do something stupid in front of other children*  *2.My child worries when they go to bed at night*  *3.My child complains of their heart suddenly starting to beat too quickly for no reason*  *4.Do your child's fears, worries or anxiety make these things difficult for your everyday life in the following areas? a. your relationships with family or friends^b^*  *5.My child would feel scared if they had to stay away from home overnight*  *6.Do fears, worries or anxiety upset or distress your child?^a^*  *7.My child has to keep checking that they have done things right (like the switch is off, or the door is locked)* *^a,b^* | 0.8755 |
| Teacher-report | 1 | *1. Worries that bad things will happen to them*  *2. Complains of their heart suddenly starting to beat too quickly for no reason*  *3. Worries they might say or do something stupid in front of other children*  *4. Do this child's fears, worries or anxiety make things difficult for you or the class as a whole?*  *5. Talks in front of teachers, when appropriate to do so*  *6. Worries when they think they have done poorly at something* |  |
|  | 2 | *1. Do this child's fears, worries or anxiety make things difficult for you or the class as a whole?* | 0.6296 |
|  | 3 | *1. Do this child's fears, worries or anxiety make things difficult for you or the class as a whole?*  *2. Do fears, worries or anxiety cause problems for this child?* *^a^*  *3. Talks in front of other children in the class, when appropriate to do so* *^a^* | 0.7614 |

1. Initial model including the items with the highest loading for each factor.

2. Model including items retained after stepwise backward elimination.

3. Model including items retained after stepwise backward elimination, together with items added following checking of remaining items

a=additional item added following checking of remaining items. b=considered replacing item

Table S10 Exploratory factor analysis of 53 child-report items assessing anxiety symptoms: Oblique rotated factor loadings for 5 salient factors

| Item | Factor 1 | Factor 2 | Factor 3 | Factor 4 | Factor 5 |
| --- | --- | --- | --- | --- | --- |
| worry_c | 0.470*^a^ | 0.153* | 0.011 | 0.310* | -0.090* |
| stom_c | 0.461* ^a^ | -0.032 | 0.135* | 0.132 | -0.105* |
| afraid_c | 0.447* ^a^ | 0.022 | -0.127* | 0.415* ^a^ | 0.03 |
| alone_c | 0.689* ^a^ | -0.02 | -0.105 | 0.027 | -0.032 |
| test_c | 0.334* | 0.450* ^a^ | 0.029 | -0.092 | 0.067 |
| nerves_c | 0.479* ^a^ | 0.087 | -0.04 | 0.249* | -0.014 |
| toilet_c | 0.389* | 0.175* | 0.222* | -0.065 | 0.009 |
| **awayp_c** | **0.777*^b^** | -0.077 | -0.019 | 0.072 | -0.016 |
| fool_c | 0.084 | 0.634* ^a^ | -0.119* | 0.187* | 0.045 |
| work_c | 0.103 | 0.615* ^a^ | 0.018 | 0.091 | 0.036 |
| talkch_c | 0.068 | 0.147* | 0.03 | -0.041 | 0.694* ^a^ |
| awffam_c | 0.191* | -0.008 | 0.113 | 0.560* ^a^ | 0.048 |
| breath_c | 0.181* | -0.026 | 0.467* ^a^ | 0.258* | 0.155* |
| check_c | 0.027 | 0.094 | 0.591* ^a^ | -0.011 | 0.016 |
| sleep_c | 0.587* ^a^ | -0.166* | -0.029 | 0.336* | 0.116* |
| school_c | 0.455* ^a^ | 0.126* | 0.145* | 0.191* | 0.078 |
| silly_c | 0.034 | 0.021 | 0.323* | 0.430* | -0.155* |
| heart_c | 0.094 | 0.048 | 0.504* ^a^ | 0.11 | -0.091 |
| trembl_c | 0.159 | -0.088 | 0.586* ^a^ | 0.171 | 0.045 |
| **bad_c** | 0.089 | 0.064 | -0.022 | **0.828* ^b^** | 0.084 |
| shaky_c | 0.242* | 0.146* | 0.427* ^a^ | 0.044 | 0 |
| specl_c | 0.101 | 0.053 | 0.448* ^a^ | 0.179 | 0.056 |
| stupid_c | -0.028 | 0.625* ^a^ | 0.011 | 0.338* | 0.021 |
| travl_c | 0.337* | 0.104 | 0.335* | 0.048 | 0.024 |
| othink_c | -0.085 | 0.612* ^a^ | -0.028 | 0.412* ^a^ | -0.009 |
| crowd_c | 0.349* | 0.125 | 0.419* ^a^ | -0.073 | 0.053 |
| scared_c | -0.053 | -0.017 | 0.648* ^a^ | 0.23 | 0.004 |
| dizzy_c | 0.205* | 0.534* ^a^ | 0.052 | -0.019 | 0.320* |
| front_c | 0.308* | -0.018 | 0.175* | 0.399* | -0.017 |
| **wakeup_c** | -0.075 | -0.04 | **0.689* ^b^** | 0.360* | -0.038 |
| hrt_nr_c | 0.274* | -0.02 | 0.248* | 0.481* ^a^ | 0.035 |
| wscare_c | 0.355* | 0.144* | 0.347* | -0.143* | -0.002 |
| small_c | -0.042 | 0.119 | 0.631* ^a^ | -0.031 | 0.022 |
| repeat_c | 0.03 | 0.076 | 0.249* | 0.500* | -0.166* |
| bother_c | -0.019 | -0.046 | 0.553* ^a^ | 0.207* | 0.074 |
| stpbad_c | 0.618* ^a^ | 0.037 | 0.053 | -0.022 | -0.008 |
| awayh_c | 0.021 | 0.566* ^a^ | 0.098 | 0.143 | -0.216* |
| poorly_c | 0.239* | 0.416* ^a^ | 0.099 | 0.109 | -0.176* |
| **angry_c** | -0.142* | **0.736*^b^** | -0.108* | 0.358* | 0.013 |
| **flish_c** | -0.076 | 0.042 | 0.061 | 0.152 | **0.736*^b^** |
| talkt_c | 0.096 | 0.06 | 0.036 | 0.822* ^a^ | 0.086 |
| awfslf_c | 0.073 | 0.667* ^a^ | 0.153* | 0.005 | -0.082* |
| mistak_c | 0.001 | 0.298* | 0.135* | 0.522* ^a^ | -0.121* |
| happen_c | -0.077 | 0.029 | 0.105 | 0.668* ^a^ | -0.078 |
| death_c | 0.513* ^a^ | -0.024 | -0.045 | 0.416* ^a^ | -0.007 |
| wbed_c | 0.024 | -0.025 | 0.05 | 0.148 | 0.457* ^a^ |
| qsttch_c | 0.299* | 0.117* | 0.171* | 0.418* ^a^ | -0.154* |
| stpwry_c | 0.322* | 0.393* | 0.149* | -0.08 | 0.017 |
| newppl_c | 0.026 | 0.721* ^a^ | -0.042 | 0.205* | 0.027 |
| embras_c | 0.194* | 0.170* | 0.198* | 0.392* | -0.136* |
| w_age_c | 0.294* | 0.160* | 0.037 | 0.281* | -0.105* |
| avoid_c | 0.362* | 0.198* | 0.116 | 0.092 | -0.147* |
| else_c | 0.265* | 0.110* | 0.194* | 0.376* | -0.096* |

*p<0.05

a factor loading >0.4

b highest loading for the factor

Table S11 Exploratory factor analysis of 54 parent-report items assessing anxiety symptoms: Oblique rotated factor loadings for 6 salient factors

| Item | Factor 1 | Factor 2 | Factor 3 | Factor 4 | Factor 5 | Factor 6 |
| --- | --- | --- | --- | --- | --- | --- |
| worry_p | -0.094 | 0.812* ^a^ | 0.057 | -0.100* | 0.160* | -0.027 |
| stom_p | -0.03 | 0.549* ^a^ | 0.02 | -0.077 | 0.243* | 0.029 |
| afraid_p | 0.053 | 0.668* ^a^ | 0.006 | 0.043 | 0.046 | 0.12 |
| alone_p | -0.026 | 0.459* ^a^ | 0.062 | -0.071 | 0.073 | 0.277* |
| test_p | -0.082 | 0.347* | 0.216* | 0.049 | 0.462* ^a^ | -0.05 |
| nerves_p | -0.177* | 0.688* ^a^ | 0.182* | -0.034 | 0.239* | -0.006 |
| toilet_p | 0.002 | 0.444* ^a^ | 0.039 | 0.151* | 0.046 | 0.200* |
| awayp_p | 0.054 | 0.489* ^a^ | -0.032 | -0.122* | 0.106 | 0.463* ^a^ |
| fool_p | -0.051 | 0.018 | 0.822* ^a^ | 0.124* | -0.019 | 0.143* |
| work_p | -0.038 | 0.240* | 0.457* ^a^ | 0.034 | 0.458* ^a^ | -0.166* |
| talkch_p | 0.016 | 0.048 | 0.069 | 0.766* ^a^ | 0.1 | 0.076 |
| awffam_p | 0.597*^a^ | 0.028 | 0.205* | -0.340* | 0.092* | 0.111 |
| breath_p | 0.633* ^a^ | 0.024 | 0.006 | -0.034 | 0.379* | 0.061 |
| check_p | 0.714* ^a^ | 0.029 | -0.045 | 0.11 | 0.035 | -0.188* |
| sleep_p | 0.098 | 0.555* ^a^ | -0.088 | 0.05 | -0.09 | 0.313* |
| school_p | 0.066 | 0.630* ^a^ | 0.019 | 0.146* | 0.173* | -0.01 |
| silly_p | 0.479* ^a^ | 0.406* ^a^ | 0.141* | -0.045 | -0.196* | -0.047 |
| heart_p | 0.566* ^a^ | -0.04 | 0.017 | 0.079 | 0.580* ^a^ | 0.063 |
| trembl_p | 0.508* ^a^ | 0.182 | -0.044 | 0.161* | 0.242* | 0.043 |
| bad_p | 0.501* ^a^ | 0.320* | 0.192* | -0.156* | -0.072 | 0.039 |
| shaky_p | 0.343* | 0.157 | 0.067 | 0.006 | 0.324* | 0.056 |
| specl_p | 0.705* ^a^ | 0.003 | 0.031 | 0.082 | 0.016 | -0.021 |
| **stupid_p** | 0.118* | -0.094* | **0.903* ^a, b^** | 0.143* | -0.006 | -0.004 |
| travl_p | 0.153 | 0.316* | 0 | -0.001 | 0.113 | 0.300* |
| othink_p | -0.021 | 0.133* | 0.795* ^a^ | -0.04 | -0.003 | 0.026 |
| crowd_p | 0.183* | 0.285* | 0.125 | 0.157* | 0 | 0.349* |
| scared_p | 0.389* | 0.614* ^a^ | -0.124* | 0.043 | 0.034 | 0.07 |
| dizzy_p | 0.502* ^a^ | 0.184 | -0.128 | -0.046 | 0.341* | 0.115 |
| front_p | -0.022 | 0.116 | 0.420* ^a^ | 0.448* ^a^ | 0.179* | 0.162* |
| wakeup_p | 0.206* | 0.611* ^a^ | -0.106 | 0.036 | -0.012 | 0.134 |
| **hrt_nr_p** | 0.676* ^a^ | -0.048 | -0.04 | 0.076 | **0.635* ^a, b^** | 0.119 |
| wscare_p | 0.338* | 0.594* ^a^ | -0.052 | 0.137* | -0.026 | 0.069 |
| small_p | 0.273* | -0.062 | 0.161* | 0.064 | 0.034 | 0.420* ^a^ |
| repeat_p | 0.676* ^a^ | 0.067 | -0.061 | 0.211* | -0.057 | -0.158 |
| bother_p | 0.551* ^a^ | 0.395* | 0.13 | -0.072 | -0.168* | -0.091 |
| **stpbad_p** | **0.876* ^a, b^** | -0.098 | 0.038 | 0.183* | -0.04 | -0.132 |
| **awayh_p** | 0.028 | 0.376* | 0.091 | -0.025 | 0.083 | **0.503* ^a, b^** |
| poorly_p | 0.230* | 0.061 | 0.590* ^a^ | -0.05 | 0.383* | -0.211* |
| angry_p | 0.209* | 0.213* | 0.409* ^a^ | -0.091* | 0.220* | -0.013 |
| flish_p | 0.035 | 0.02 | 0.848* ^a^ | 0.037 | -0.033 | 0.119* |
| talkt_p | 0.023 | -0.014 | 0.057 | **0.832* ^a, b^** | 0.003 | 0.02 |
| awfp_p | 0.631* ^a^ | 0.098 | 0.214* | -0.323* | 0.003 | 0.156* |
| mistak_p | 0.255* | -0.019 | 0.646* ^a^ | -0.031 | 0.349* | -0.221* |
| happen_p | 0.299* | 0.536* ^a^ | 0.160* | -0.168* | 0.078* | -0.121* |
| death_p | 0.430* ^a^ | 0.115 | 0.219* | -0.184* | -0.047 | -0.064 |
| wbed_p | 0.192* | 0.710* ^a^ | -0.058 | -0.038 | -0.123* | 0.118 |
| qsttch_p | 0.095 | 0.059 | 0.151* | 0.567* ^a^ | 0.055 | -0.018 |
| stpwry_p | 0.173* | 0.734* ^a^ | 0.094* | -0.015 | 0.008 | -0.067 |
| newppl_p | -0.039 | 0.309* | 0.352* | 0.178* | 0.032 | 0.285* |
| embras_p | 0.016 | 0.006 | 0.897* ^a^ | 0.107 | -0.162* | 0.184* |
| w_age_p | 0.039 | 0.940* ^a^ | 0.015 | 0.166* | 0.009 | -0.188* |
| **w_sit_p** | -0.021 | **0.958* ^a, b^** | 0.032 | 0.198* | -0.034 | -0.201* |
| avoid_p | 0.022 | 0.566* ^a^ | 0.146* | 0.158* | -0.08 | 0.166* |
| else_p | 0.071 | 0.498* ^a^ | -0.019 | 0.023 | -0.074 | 0.288* |

*p<0.05

a factor loading >0.4

b highest loading for the factor

Table S12 Exploratory factor analysis of 50 teacher-report items assessing anxiety symptoms: Oblique rotated factor loadings for 5 salient factors

| Item | Factor 1 | Factor 2 | Factor 3 | Factor 4 | Factor 5 |
| --- | --- | --- | --- | --- | --- |
| worry_t | 0.037 | 0.553* ^a^ | 0.174* | 0.331* | -0.057 |
| stom_t | 0.292* | 0.456* ^a^ | 0.141* | 0.046 | -0.037 |
| afraid_t | 0.351* | 0.438* ^a^ | 0.077 | 0.171* | -0.044 |
| test_t | 0.148* | 0.227* | 0.267* | 0.417* ^a^ | 0.024 |
| nerves_t | 0.201* | 0.249* | 0.265* | 0.365* | 0.091* |
| toilet_t | 0.442*^a^ | 0.282* | 0.048 | 0.015 | 0.313* |
| awayp_t | 0.364* | 0.462* ^a^ | 0.199* | -0.039 | -0.031 |
| fool_t | 0.051 | 0.054 | 0.746* ^a^ | 0.119 | 0.095* |
| work_t | 0.023 | 0.028 | 0.321* | 0.643* ^a^ | -0.006 |
| talkch_t | -0.001 | 0.025 | 0.224* | -0.07 | 0.745* ^a^ |
| awffam_t | 0.281 | 0.694* ^a^ | -0.013 | -0.198* | 0.011 |
| breath_t | 0.786* ^a^ | 0.125 | -0.214* | 0.165 | -0.028 |
| check_t | 0.262* | -0.004 | 0.053 | 0.364* | 0.105 |
| school_t | 0.315* | 0.475* ^a^ | 0.087 | 0.072 | 0.072 |
| **silly_t** | 0.01 | **0.871* ^a, b^** | -0.169* | 0.161 | -0.018 |
| heart_t | 0.947* ^a^ | -0.159 | 0.02 | 0.126 | -0.053 |
| trembl_t | 0.526* ^a^ | -0.024 | 0.011 | 0.442* | 0.186* |
| bad_t | 0.257* | 0.725* ^a^ | 0.004 | -0.039 | -0.038 |
| shaky_t | 0.605* ^a^ | 0.055 | -0.025 | 0.376* | 0.059 |
| specl_t | 0.414* ^a^ | 0.634* ^a^ | -0.277 | -0.053 | 0.135 |
| **stupid_t** | -0.096 | -0.021 | **0.879* ^a, b^** | 0.039 | 0.068 |
| travl_t | 0.655* ^a^ | -0.003 | 0.418* ^a^ | -0.055 | -0.156 |
| othink_t | -0.247* | 0.188* | 0.762* ^a^ | 0.153 | -0.109* |
| crowd_t | 0.597* ^a^ | 0.222 | 0.234* | -0.015 | 0.183* |
| scared_t | 0.431* ^a^ | 0.417* ^a^ | 0.016 | 0.172 | 0.053 |
| dizzy_t | 0.869* ^a^ | -0.023 | -0.272* | 0.092 | -0.07 |
| front_t | -0.046 | 0.034 | 0.576* ^a^ | 0.035 | 0.425* ^a^ |
| **hrt_nr_t** | **1.124* ^a, b^** | -0.290* | -0.021 | 0.148 | -0.007 |
| wscare_t | 0.479* ^a^ | 0.296* | 0.191* | 0.046 | 0.115 |
| small_t | 0.776* ^a^ | 0.094 | 0.212* | -0.135 | 0.065 |
| repeat_t | 0.456* ^a^ | 0.058 | -0.046 | 0.291* | 0.321* |
| bother_t | 0.026 | 0.819* ^a^ | -0.087 | 0.045 | -0.044 |
| stpbad_t | 0.498* ^a^ | 0.178 | -0.048 | 0.221 | 0.274* |
| awayh_t | 0.432* ^a^ | 0.253 | 0.307* | -0.166* | -0.079 |
| **poorly_t** | 0.044 | -0.058 | 0.239 | **0.771* ^a, b^** | -0.172* |
| angry_t | 0.047 | 0.079 | 0.286* | 0.581* ^a^ | -0.149* |
| flish_t | -0.023 | -0.109 | 0.851* ^a^ | 0.128 | 0.046 |
| **talkt_t** | 0.044 | -0.087 | 0.05 | 0.033 | **0.908* ^a, b^** |
| awfslf_t | 0.203 | 0.745* ^a^ | 0.007 | -0.01 | -0.079 |
| mistak_t | 0.022 | 0.017 | 0.283* | 0.680* ^a^ | -0.123* |
| happen_t | 0.13 | 0.509* ^a^ | 0.195* | 0.283* | -0.063 |
| death_t | 0.207 | 0.542* ^a^ | -0.03 | -0.173* | -0.044 |
| qsttch_t | 0 | -0.024 | 0.038 | -0.047 | 0.827* ^a^ |
| stpwry_t | -0.013 | 0.647* ^a^ | 0.059 | 0.427* ^a^ | -0.053 |
| newppl_t | 0.441* ^a^ | 0.113 | 0.507* ^a^ | -0.124 | 0.082 |
| embras_t | 0.139 | -0.094 | 0.877* ^a^ | 0.036 | 0.07 |
| w_age_t | -0.241* | 0.829* ^a^ | 0.016 | 0.464* ^a^ | 0.069 |
| w_sit_t | -0.293* | 0.819* ^a^ | -0.003 | 0.532* ^a^ | 0.068 |
| avoid_t | 0.218* | 0.361* | 0.351* | 0.135* | 0.137* |
| else_t | 0.471* ^a^ | 0.283* | 0.170* | -0.003 | -0.051 |

*p<0.05

a factor loading >0.4

b highest loading for the factor

Table S13 Summary of factors identified in exploratory factor analysis (symptom items)

| Child-report | **Factor 1** – ‘generalised anxiety’ (explains 42.2% of the variance)  Highest loading item (0.777): *I worry about being away from my parents*  **Factor 2** – 'social anxiety’ (explains 4.7% of the variance)  Highest loading item (0.736): *I feel worried when I think someone is angry with me*  **Factor 3** – 'panicky symptoms’ (explains 4.1% of the variance)  Highest loading item (0.689): *I wake up feeling scared*  **Factor 4** – 'worry’ (explains 3.7% of the variance)  Highest loading item (0.828): *I worry that something bad will happen to me*  **Factor 5** – 'performance anxiety’ (explains 3.0% of the variance)  Highest loading item (0.736): *I worry I might look foolish* |
| --- | --- |
| Parent-report | **Factor 1** – ‘generalised anxiety’. (explains 43.3% of the variance)  Highest loading item (0.876): *My child has to do certain things in just the right way to stop bad things happening*  **Factor 2** – ‘worry’ (explains 7.1% of the variance).  Highest loading item (0.958): *My child worries about things more than other children in a similar situation*  **Factor 3** – ‘social anxiety’ (explains 5.1% of the variance).  Highest loading item (0.903): *My child worries they might say or do something stupid in front of other children*  **Factor 4** – ‘talking in class’ (explains 3.8% of the variance).  Highest loading item (0.832): *My child talks in front of teachers*  **Factor 5** – ‘panicky’ (explains 3.2% of the variance).  Highest loading item (0.635): *My child complains of their heart suddenly starting to beat too quickly for no reason*  **Factor 6** – ‘separation anxiety/away from home’ (explains 3.0% of the variance).  Highest loading item (0.503): *My child would feel scared if they had to stay away from home overnight* |
| Teacher-report | **Factor 1** – ‘physical/observable symptoms’ (explains 49.2% of the variance)  Highest loading item (1.124): *Complains of their heart suddenly starting to beat too quickly for no reason*  **Factor 2** – ‘worry’ (explains 9.6% of the variance).  Highest loading item (0.871): *Can't seem to get bad or silly thoughts out of their head*  **Factor 3** – ‘social anxiety’ (explains 6.0% of the variance).  Highest loading item (0.879): *Worries they might say or do something stupid in front of other children*  **Factor 4** – ‘school performance worries’ (explains 4.5% of the variance).  Highest loading item (0.771): *Worries when they think they have done poorly at something*  **Factor 5** – ‘talking in class’ (0.908) (explains 3.3% of the variance).  Highest loading item: *Talks in front of teachers, when appropriate to do so* |

Tables S14 Indicators of fit for exploratory factor analysis models (symptom items)

| Analysis | N | Number of items | Number of factors | Chi-squared (df) | RMSEA (90% CI) | CFI | TLI | SRMR |
| --- | --- | --- | --- | --- | --- | --- | --- | --- |
| Child-report | 520 | 53 | 5 | 1939.45 (df = 1123) | 0.037 (90% CI: 0.035 to 0.040) | 0.971 | 0.964 | 0.040 |
| Parent-report | 568 | 54 | 6 | 2228.6 (df = 1122) | 0.042 (90% CI: 0.039 to 0.044) | 0.969 | 0.961 | 0.045 |
| Teacher-report | 464 | 50 | 5 | 1396.8 (df = 985) | 0.030 (90% CI: 0.026 to 0.034) | 0.984 | 0.981 | 0.057 |

RMSEA – Root mean square error of approximation

CFI – Comparative Fit Index

TLI – Tucker-Lewis Index

SRMR – Standardised Root Mean Square Residual

Table S15 Identifying best predictors of anxiety disorder status: Backward elimination stepwise logistic regression (symptom items)

| Reporter | Model | Items | Area Under the Curve |
| --- | --- | --- | --- |
| Child-report | 1 | *1.I worry about being away from my parents*  *2.I feel worried when I think someone is angry with me*  3. *I wake up feeling scared*  4. *I worry that something bad will happen to me*  *5.I worry I might look foolish* |  |
|  | 2 | *1. I worry about being away from my parents*  *2. I wake up feeling scared* | 0.6536 |
|  | 3 | *1. I worry about being away from my parents*  *2. I wake up feeling scared*  3. *I worry about things more than other children my age* ^a^  4. *I have trouble going to school in the mornings because I feel nervous or afraid* ^a^  5. *I feel afraid if I have to talk in front of my class* ^a^  6. *I try to avoid something because it scares me^a^* | 0.7897 |
| Parent-report | 1 | *1.My child has to do certain things in just the right way to stop bad things happening*  *2.My child worries about things more than other children in a similar situation*  *3.My child worries they might say or do something stupid in front of other children*  *4. My child talks in front of teachers*  *5. My child complains of their heart suddenly starting to beat too quickly for no reason*  *6. My child would feel scared if they had to stay away from home overnight* |  |
|  | 2 | *1.My child has to do certain things in just the right way to stop bad things happening*  *2.My child worries about things more than other children in a similar situation*  *3. My child complains of their heart suddenly starting to beat too quickly for no reason*  *4. My child would feel scared if they had to stay away from home overnight* | 0.8283 |
|  | 3 | *1.My child has to do certain things in just the right way to stop bad things happening^b^*  *2.My child worries about things more than other children in a similar situation^b^*  *3. My child complains of their heart suddenly starting to beat too quickly for no reason*  *4. My child would feel scared if they had to stay away from home overnight*  *5. My child can't seem to get bad or silly thoughts out of their head^a^*  *6. My child has to keep checking that they have done things right (like the switch is off, or the door is locked) ^a, b^*  *7. My child is scared if they have to sleep on their own^a^* | 0.8719 |
| Teacher-report | 1 | *1.Complains of their heart suddenly starting to beat too quickly for no reason*  2. *Can't seem to get bad or silly thoughts out of their head*  *3. Worries they might say or do something stupid in front of other children*  *4. Worries when they think they have done poorly at something*  *5. Talks in front of teachers, when appropriate to do so* |  |
|  | 2 | 1. *Can't seem to get bad or silly thoughts out of their head*  *2. Worries when they think they have done poorly at something*  *3. Talks in front of teachers, when appropriate to do so* | 0.6698 |
|  | 3 | *1. Can't seem to get bad or silly thoughts out of their head*  *2. Worries when they think they have done poorly at something*  *3. Talks in front of teachers, when appropriate to do so*  *4. Worries about things ^a^*  *5. Talks in front of other children in the class, when appropriate to do so* ^a^ | 0.7498 |

1. Initial model including the items with the highest loading for each factor.

2. Model including items retained after stepwise backward elimination.

3. Model including items retained after stepwise backward elimination, together with items added following checking of remaining items

a=additional item added following checking of remaining items. b=considered replacing item

Table S16 Identifying best predictors of anxiety disorder status: Backward elimination stepwise logistic regression (impact items)

| Reporter | Model | Items |
| --- | --- | --- |
| Child-report | 1 | *1. Do fears or worries cause problems for you?*  *2. Do fears or worries upset you?*  *3. Do fears or worries stop you from doing things?*  *4. Do your fears or worries make things difficult for people around you (e.g. family, friends, teachers)?*  *5. Would you like some help with fears or worries?* |
|  | 2 | *1. Do fears or worries cause problems for you?*  *2. Would you like some help with fears or worries?* |
| Parent-report | 1 | *1.Do fears, worries or anxiety cause problems for your child?*  *2. Do fears, worries or anxiety upset or distress your child?*  *3. Do fears, worries or anxiety stop your child from doing things?*  *4. Do your child's fears, worries or anxiety make things difficult for your family as a whole?*  *5. Do you think your child would benefit from some support with fears, worries or anxiety?*  *6. Do you think you would benefit from some support to help your child overcome difficulties with fears, worries or anxiety?* |
|  | 2 | *1. Do fears, worries or anxiety upset or distress your child?*  *2. Do your child's fears, worries or anxiety make things difficult for your family as a whole?* |
| Teacher-report | 1 | *1.Do fears, worries or anxiety cause problems for this child?*  *2.Do fears, worries or anxiety upset or distress this child?*  *3.Do fears, worries or anxiety stop this child from doing things?*  *4.Do this child's fears, worries or anxiety make things difficult for you or the class as a whole?*  *5.Do you think this child would benefit from some support with fears, worries or anxiety?* |
|  | 2 | *1.Do fears, worries or anxiety upset or distress this child?*  *2.Do fears, worries or anxiety stop this child from doing things?* |

1. Initial model

2. Model including items retained after stepwise backward elimination.

Table S17 Summary of the ability of the candidate parent-report measures to discriminate between children with and with-out anxiety disorders among subgroups according to age, gender and ethnic background

|  | Candidate measure 1, version A (symptoms and impact) | Candidate measure 1, version B (symptoms and impact) | Candidate measure 2, version A  (2 stage) | Candidate measure 2, version B  (2 stage) | Candidate measure 3  (impact only) |
| --- | --- | --- | --- | --- | --- |
| Year 4  N  Sensitivity  Specificity | 151  76.5% (26/34)  69.2% (81/117) | 151  61.8% (21/34)  81.2% (95/117) | 147  68.8% (22/32)  81.7% (94/115) | 149  70.6% (24/34)  82.6% (95/115) | 153  71.4% (25/35)  82.2% (97/118) |
| Year 5  N  Sensitivity  Specificity | 169  92.3% (36/39)  73.9% (96/130) | 169  89.7% (35/39)  82.3% (107/130) | 169  87.2% (34/39)  79.2% (103/130) | 169  87.5% (35/40)  78.3% (101/129) | 172  90.2% (37/41)  76.3% (100/131) |
| Year 6  N  Sensitivity  Specificity | 135  73.3% (22/30)  76.2% (80/105) | 135  71.0% (22/31)  83.7% (87/104) | 134  72.4% (21/29)  86.7% (91/105) | 134  63.3% (19/30)  84.6% (88/104) | 135  64.5% (20/31)  80.8% (84/104) |
|  |  |  |  |  |  |
| Female  N  Sensitivity  Specificity | 237  79.6% (43/54)  72.1% (132/183) | 237  74.6% (41/55)  83.5% (152/182) | 236  73.6% (39/53)  82.0% (150/183) | 236  78.2% (43/55)  82.9% (150/181) | 241  79.0% (45/57)  80.4% (148/184) |
| Male  N  Sensitivity  Specificity | 218  83.7% (41/49)  74.0% (125/169) | 218  75.5% (37/49)  81.1% (137/169) | 214  80.9% (38/47)  82.6% (138/167) | 216  71.4% (35/49)  80.2% (134/167) | 219  74.0% (37/50)  78.7% (133/169) |
|  |  |  |  |  |  |
| White British  N  Sensitivity  Specificity | 371  81.3% (74/91)  73.6% (206/280) | 371  73.9% (68/92)  82.1% (229/279) | 367  76.4% (68/89)  81.7% (227/278) | 369  73.9% (68/92)  80.1% (222/277) | 376  75.8% (72/95)  77.6% (218/281) |
| Other ethnic background  N  Sensitivity  Specificity | 84  83.3% (10/12)  70.8% (51/72) | 84  83.3% (10/12)  83.3% (60/72) | 83  81.8% (9/11)  84.7% (61/72) | 83  83.3% (10/12)  87.3% (62/71) | 84  83.3% (10/12)  87.5% (63/72) |
